# Effectiveness of self-management interventions in inflammatory arthritis: a systematic review informing the 2021 EULAR recommendations for the implementation of self-management strategies in patients with inflammatory arthritis

**DOI:** 10.1136/rmdopen-2021-001647

**Published:** 2021-05-28

**Authors:** Andréa Marques, Eduardo Santos, Elena Nikiphorou, Ailsa Bosworth, Loreto Carmona

**Affiliations:** 1 Rheumatology Department, Centro Hospitalar e Universitário de Coimbra EPE, Coimbra, Portugal; 2 Higher School of Nursing of Coimbra, Health Sciences Research Unit: Nursing, Coimbra, Portugal; 3 Centre for Rheumatic Diseases, King College London, London, UK; 4 National Rheumatoid Arthritis Society, Littlewick Green, UK; 5 Instituto de Salud Musculoesquelética, Madrid, Spain

**Keywords:** arthritis, rheumatoid, arthritis, psoriatic, spondylitis, ankylosing, outcome assessment, health care, inflammation

## Abstract

**Objective:**

To perform a systematic review (SR) on the effectiveness of self-management interventions, in order to inform the European League Against Rheumatism Recommendations for its implementation in patients with inflammatory arthritis (IA).

**Methods:**

The SR was conducted according to the Cochrane Handbook and included adults (≥18 years) with IA. The search strategy was run in Medline through PubMed, Embase, Cochrane Library, CINAHL Plus with Full Text, and PEDro. The assessment of risk of bias, data extraction and synthesis were performed by two reviewers independently. A narrative Summary of Findings was provided according to the Grading of Recommendations, Assessment, Development and Evaluation.

**Results:**

From a total 1577 references, 57 were selected for a full-text review, and 32 studies fulfilled the inclusion criteria (19 randomised controlled trials (RCTs) and 13 SRs). The most studied self-management components were specific interactive disease education in ten RCTs, problem solving in nine RCTs, cognitive–behavioural therapy in eight RCTs, goal setting in six RCTs, patient education in five RCTs and response training in two RCTs. The most studied interventions were multicomponent or single exercise/physical activity in six SRs, psychosocial interventions in five SRs and education in two SRs. Overall, all these specific components and interventions of self-management have beneficial effects on IAs-related outcomes.

**Conclusions:**

The findings confirm the beneficial effect of the self-management interventions in IA and the importance of their implementation. Further research should focus on the understanding that self-management is a complex intervention to allow the isolation of the effectiveness of its different components.

**Key messages**

**What is already known about this subject?**

- Interventions which aim to strengthen self-management skills of people with inflammatory arthritis are complex.

**What does this study add?**

- This systematic review underscores the need for a set of critical outcomes for self-management strategies.
- It highlights the beneficial effects of different components of self-management, such as specific interactive disease education, problem solving, cognitive–behavioural therapy, goal setting, patient education, response training, and globally, multicomponent or single exercise/physical activity and psychosocial interventions on some patient outcomes, including self-efficacy.
- However, evidence of the effectiveness of self-management in outcome results in patients with inflammatory arthritis is lacking.

**How might this impact on clinical practice or further developments?**

- This systematic review highlights the importance of incorporating self-management interventions in routine clinical care.
- Future research should explore which intervention components contribute most to achieving better critical outcomes.

## Introduction

Inflammatory arthritis (IA), including rheumatoid arthritis (RA), psoriatic arthritis (PsA), ankylosing spondylitis (AS) and axial spondyloarthritis (ax-SpA) or unspecified polyarthritis (UA), is the chronic conditions with a pervasive impact on daily self-care and quality of life.^1^ An essential aspect of adjusting to IA is the ability to understand the disease and deal with the practical, physical and psychological impacts that come along with it.^2^ This goes beyond drug therapy and implies the recognition that the diagnosis of IA is life-changing, and that the ability to self-manage is crucial.^3^

Self-management, unlike the traditional medical model, emphasises the importance of interactive, collaborative care between the patient and the healthcare professional rather than one-way, passive care from expert to patient. Although several educational materials and resources may be available for self-management of IA, these are often underused and not always incorporated in the routine care. Time pressures, limited healthcare services, the perceived lack of evidence but also the lack of knowledge of healthcare professionals as to who is available and what resources are available to best address self-management aspects of care, are recognised obstacles to providing the necessary support in a sensitive and patient-centred manner.^4^

Several European Alliance of Associations for Rheumatology (EULAR) recommendations for the management of specific RMDs have highlighted the importance of self-management to achieve the desired effect of interventions.^5–9^ However, these recommendations do not orient clinicians and healthcare professionals on how to support patients to self-management, acquire self-management skills and make necessary behavioural changes.

In order to inform the task force responsible for the 2021 EULAR Recommendations for the implementation of self-management strategies in patients with IA, we performed a systematic review (SR) that aimed to identify the best evidence on the effectiveness of self-management interventions targeting IA and to describe their components.

## Methods

This SR was conducted according to the Cochrane Handbook^10^ and reported following the Preferred Reporting Items for Systematic Reviews and Meta-Analyses guidelines.^11^

The steering group of the EULAR task force (AM, ES, EN, AB and LC) established and followed the SR protocol, which was not registered, but is available on request. The outlined research questions, as approved by the entire task force at the first meeting, were: which self-management interventions are effective in IA? Which are the components of effective interventions? Who are the professionals who deliver these effective interventions? These questions were framed and structured according to the EULAR standardised operating procedures^12^ using the ‘Patients, Intervention, Comparator or Control, Outcome, Type of study format’.

### Participants

A study was eligible for inclusion if the participants included were adults (≥18 years) with IA (specifically, RA, PsA, AS, ax-SpA or UA). To maximise precision, only studies in which patients were formally diagnosed with IA or who satisfied current disease criteria, were included.^13–16^ Studies focusing on the information regarding patients with other concomitant diseases, whether these were rheumatic or not, were excluded from the synthesis.

### Interventions

With regards to eligible interventions, these had to be defined explicitly as ‘self-management’, in other words, the individual patient ability and competence regarding the management of symptoms, treatment, physical and psychosocial consequences and the lifestyle changes inherent in living with a chronic condition^17^; or they had to include at least one component from each of the following: biological, psychological and social management. Interventions must consist of disease information, medication management, management of the physical activity, disease-related problem solving, cognitive symptom management, management of emotions, communication skills and use of community resources.^18^ Additionally, these interventions should be promoted by, or result from interaction with a programme leader who is a health professional. They may be delivered face to face or online, with direct or indirect trained support provided.

### Comparator or control

The comparator was placebo or usual care (standard care).

### Context

There were no contextual constraints in this SR.

### Outcomes

Concerning outcomes, the core concept in self-management is the realisation of self-efficacy; that is, confidence in oneself to carry out the required behaviour to acquire the desired goal.^19^ We accepted other patient-reported outcome measures that were quantitative measures of the impact of the disease, such as pain, functional disability, fatigue, emotional well-being, sleep, coping and physical well-being (eg, Visual Analogue Scale, Health Assessment Questionnaire, Functional Assessment of Chronic Illness Therapy, Rheumatoid Arthritis Impact of Disease).^20 21^ Health-related quality of life (eg, 36-Item Short Form Survey, EuroQol-5 Dimension).^22^ Self-efficacy should be measured by a validated tool (eg, General Self-Efficacy Scale or Stanford Self-Efficacy Scale].

### Type of study

Eligible designs were only SR and randomised controlled trials (RCTs) or controlled clinical trials because they are the most robust designs and represent the highest evidence.

### Search strategy and study selection

A search strategy was run in Medline through PubMed, Embase, Cochrane Library, CINAHL Plus with Full Text, and PEDro from 20 January 2020 to 24 January 2020. Studies published in English, French, Spanish, and Portuguese language, with no restriction of the publication date, were considered for inclusion. Details on complete search strategies are provided in online supplemental material S1.

10.1136/rmdopen-2021-001647.supp1 Supplementary data

All identified citations were uploaded into an EndNote VX7 (Clarivate Analytics, Pennsylvania, USA) library and the duplicates removed. Titles and abstracts were screened by two independent reviewers (ES and AM) to assess eligibility criteria. The full articles were retrieved for all studies that met or had insufficient information to assess these inclusion criteria, and two reviewers (ES and AM) independently examined them in detail. Any disagreements that arose between the reviewers were resolved through discussion or with a third reviewer (LC).

### Assessment of risk of bias, data extraction and synthesis

Two reviewers (ES and AM) independently assessed the risk of bias of each included study using the AMSTAR2 for SR^23^ and the Cochrane Collaboration’s tool for RCT’s.^24^ Any disagreements between the reviewers were resolved through discussion, or with a third reviewer (LC).

Data were extracted from the selected reports by the same two independent reviewers (ES and AM), and disagreements were discussed until consensus was achieved, with the third reviewer (LC) involved whenever necessary. Authors of papers were contacted to request missing or additional data, where required. The overlap of original research studies included in SRs was rigorously checked to avoid double counting and expressed as percentage.

An overall assessment of the quality of the evidence for each comparison (intervention vs control) was performed using the Grading of Recommendations, Assessment, Development and Evaluation^25^ and Summary of Findings (SoF) tables were produced with the GRADEPro GDT software. A four-point rating scale was used to rate the quality of the evidence (high, moderate, low and very low), according to the following criteria: risk of bias, inconsistency, imprecision, indirectness and publication bias. A narrative SoF form was preferred due to the differences in metrics used by the included studies.

## Results

**Figure 1:**
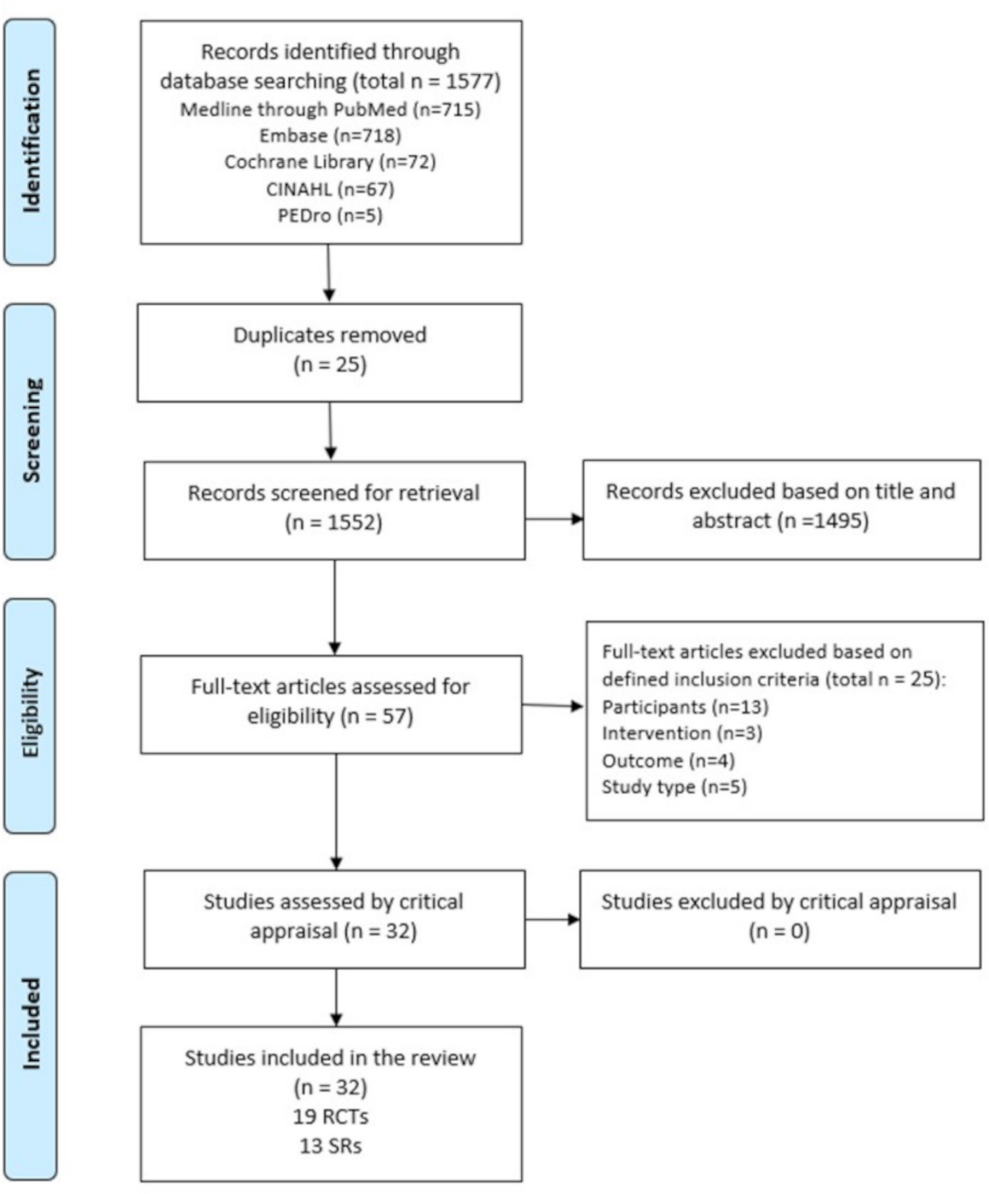
Flow chart of the study selection and inclusion process.

From a total of 1577 references, 57 were selected for a full-text review, and 32 studies fulfilled the inclusion criteria. Full-text studies that did not meet the inclusion criteria were excluded, and reasons for exclusion are provided in online supplemental material S2. Included studies were 19 RCTs and 13 SRs. As a result of the overlap, 91 RCTs (34.9%) were duplicated in the SRs. Only one author of the papers was contacted to request additional information. The results of the searches are shown in a flow diagram (figure 1).

10.1136/rmdopen-2021-001647.supp2 Supplementary data

### Methodological quality

The critical appraisal results for each of the studies are summarised in online supplemental material S3. There was agreement among the reviewers to include all the studies that were appraised. Regarding SRs, most (n=9) had moderate quality, three had high quality and only one was of low quality. This lower quality was mainly due to problems of no explicit statement that the review methods were established prior to the conducting of the review, issues in selection of the study designs, insufficient search strategy, not providing a list of excluded studies and justifying the exclusions, not reporting on the sources of funding and not investigating the publication bias. The majority of the RCTs included were of moderate to high quality, except for one, that was low. In general, all RCTs had issues with allocation concealment and blinding of participants and outcomes, which might be expected given the nature of the intervention.

10.1136/rmdopen-2021-001647.supp3 Supplementary data

### Characteristics of included studies and interventions

Study characteristics are detailed in online supplemental material S4. Regarding interventions, the most commonly studied among the 19 RCTs were specific interactive disease education (n=10),^26–35^ problem solving (n=9),^26 28 32 33 35–40^ cognitive–behavioural therapy (n=8),^32 33 38–43^ goal setting (n=6),^26 32 35–38 40^ patient education (n=5),^35–37 41 44–46^ and response training (n=2).^32 34^ Of note, several RCTs had addressed more than one intervention.

10.1136/rmdopen-2021-001647.supp4 Supplementary data

Of the 13 included SRs, the most studied interventions were multi-component or single exercise/physical activity (n=6),^47–52^ psychosocial interventions (n=5),^47 53–56^ education (n=2)^57 58^ and self-management (n=1).^59^ Studies were very heterogeneous from a methodological, clinical and even statistical point of view; thus, data pooling was not possible. Table 1 and online supplemental material S5 provide a summary on the effects of interventions per outcome.

10.1136/rmdopen-2021-001647.supp5 Supplementary data

**Table 1:** Short version of GRADE Summary of Findings

Interventions	Outcomes	Impact	Certainty of the evidence (GRADE)
Cognitive–behavioral therapy	Functional disability	Effective	⨁⨁◯◯ LOW
	Disease activity	Effective	⨁⨁◯◯ LOW
	Impairment/disability	Effective	⨁⨁⨁◯ MODERATE
	Anxiety/depression	Effective	⨁⨁◯◯ LOW
	Psychophysiological complains	Effective	⨁⨁◯◯ LOW
	Sleep problems	Effective	⨁⨁◯◯ LOW
	Pain	Effective	⨁⨁◯◯ LOW
	Self-efficacy/self-helplessness	Effective	⨁⨁⨁◯ MODERATE
	Quality of life/health status/social support	Effective	⨁⨁◯◯ LOW
	Healthcare use	No effect	⨁⨁◯◯ LOW
	Fatigue	Effective	⨁⨁⨁◯ MODERATE
Response training	Functional disability	Effective	⨁⨁⨁◯ MODERATE
	Disease activity	Effective	⨁⨁⨁◯ MODERATE
	Impairment/disability	Effective	⨁⨁⨁◯ MODERATE
	Psychophysiological complains	No effect	⨁⨁◯◯ LOW
	Pain	Effective	⨁⨁⨁◯ MODERATE
	Self-Efficacy/self-helplessness	Effective	⨁⨁⨁◯ MODERATE
	Quality of life/Health status/Social support	Effective	⨁⨁⨁◯ MODERATE
	Fatigue	Effective	⨁⨁⨁◯ MODERATE
Specific interactive disease education	Knowledge	Effective	⨁⨁⨁◯ MODERATE
	Functional disability	Effective	⨁⨁◯◯ LOW
	Disease activity	Effective	⨁⨁◯◯ LOW
	Impairment/disability	Effective	⨁⨁◯◯ LOW
	Anxiety/depression	No effect	⨁⨁⨁◯ MODERATE
	Psychophysiological complains	No effect	⨁⨁⨁◯ MODERATE
	Pain	Effective	⨁⨁◯◯ LOW
	Self-efficacy/self-helplessness	Effective	⨁⨁◯◯ LOW
	Quality of life/health status/social support	Effective	⨁⨁◯◯ LOW
	Fatigue	Effective	⨁⨁◯◯ LOW
Goal setting	Functional disability	Effective	⨁⨁◯◯ LOW
	Disease activity	Effective	⨁⨁◯◯ LOW
	Impairment/disability	Effective	⨁⨁⨁◯ MODERATE
	Anxiety/depression	Effective	⨁⨁◯◯ LOW
	Psychophysiological complains	Effective	⨁⨁◯◯ LOW
	Sleep problems	Effective	⨁⨁⨁◯ MODERATE
	Pain	Effective	⨁⨁◯◯ LOW
	Self-efficacy/self-helplessness	Effective	⨁⨁◯◯ LOW
	Quality of life/health status/social support	Effective	⨁⨁◯◯ LOW
	Fatigue	Effective	⨁⨁◯◯ LOW
Problem solving	Functional disability	Effective	⨁⨁◯◯ LOW
	Disease activity	Effective	⨁⨁◯◯ LOW
	Impairment/disability	Effective	⨁⨁◯◯ LOW
	Anxiety/depression	Effective	⨁⨁◯◯ LOW
	Psychophysiological complains	Effective	⨁⨁◯◯ LOW
	Sleep problems	Effective	⨁⨁⨁◯ MODERATE
	Pain	Effective	⨁⨁◯◯ LOW
	Self-efficacy/self-helplessness	Effective	⨁⨁◯◯ LOW
	Quality of life/health status/social support	Effective	⨁⨁◯◯ LOW
	Healthcare use	No effect	⨁⨁⨁◯ MODERATE
	Fatigue	Effective	⨁⨁◯◯ LOW
Multicomponent or single exercise/physical activity interventions	Pain	Effective	⨁⨁⨁◯ MODERATE
	Functional disability	Effective	⨁⨁◯◯ LOW
	Fatigue	Effective	⨁⨁⨁⨁ HIGH
	Patient Global Assessment	Effective	⨁⨁⨁◯ MODERATE
	BASDAI	Effective	⨁⨁◯◯ LOW
	BASFI	Effective	⨁⨁◯◯ LOW
	DAS-28	No effect	⨁◯◯◯ VERY LOW
Psychosocial interventions	Pain	Effective	⨁⨁⨁◯ MODERATE
	Functional disability	Effective	⨁⨁⨁◯ MODERATE
	Fatigue	Effective	⨁⨁⨁◯ MODERATE
	Psychological status	Effective	⨁⨁⨁◯ MODERATE
	Physical activity	Effective	⨁⨁⨁◯ MODERATE
	Depression	Effective	⨁⨁⨁◯ MODERATE
	Anxiety	Effective	⨁⨁⨁◯ MODERATE
	Tender joints	Effective	⨁⨁⨁◯ MODERATE
	Coping	Effective	⨁⨁⨁◯ MODERATE
	Self-efficacy	Effective	⨁⨁⨁◯ MODERATE
	DAS-28	No effect	⨁◯◯◯ VERY LOW
Self-management interventions	Pain	Effective	⨁⨁◯◯ LOW
	Functional disability	No effect	⨁◯◯◯ VERY LOW
Educational interventions	Pain	Effective	⨁⨁⨁◯ MODERATE
	Fatigue	Effective	⨁◯◯◯ VERY LOW
	Functional disability	Effective	⨁⨁⨁◯ MODERATE
	Joint counts	Effective	⨁⨁⨁◯ MODERATE
	Patient Global Assessment	Effective	⨁⨁⨁◯ MODERATE
	Psychological status	Effective	⨁⨁⨁◯ MODERATE
	Depression	Effective	⨁⨁⨁◯ MODERATE
	Adherence	Effective	⨁⨁⨁◯ MODERATE
	Self-efficacy	Effective	⨁⨁⨁◯ MODERATE

GRADE Working Group grades of evidence. High certainty: We are very confident that the true effect lies close to that of the estimate of the effect; Moderate certainty: We are moderately confident in the effect estimate: The true effect is likely to be close to the estimate of the effect, but there is a possibility that it is substantially different; Low certainty: Our confidence in the effect estimate is limited: The true effect may be substantially different from the estimate of the effect; Very low certainty: We have very little confidence in the effect estimate: The true effect is likely to be substantially different from the estimate of effect.

BASDAI, Bath Ankylosing Spondylitis Disease Activity Index; BASFI, Bath Ankylosing Spondylitis Functional Index; DAS-28, Disease Activity Score-28; GRADE, Grading of Recommendations, Assessment, Development and Evaluation.

## Discussion

This SR shows some beneficial effects of components of self-management, such as specific interactive disease education,^26–35^ problem solving,^26 28 32 33 35–40^ cognitive–behavioural therapy,^32 33 38–43^ goal setting,^26 32 35–38 40^ patient education^35–37 41 44–46^ and response training.^32 34^ Also, multicomponent or single exercise/physical activity,^47–52^ psychosocial interventions,^47 53–56^ education^57 58^ and self-management,^59^ in general, also corroborate this trend. Several other studies explored the effectiveness of self-management interventions in undifferentiated chronic diseases or other rheumatic diseases besides IA.^60–64^ Other outcomes, for example, disease activity, healthcare use, psychophysiological complaints, anxiety/depression and functional disability, were either controversial or had no positive effect.

The presentation of the findings by individual components of the self-management interventions was driven primarily by the heterogeneity of the interventions, which did not allow ‘points of convergence’. As shown in table 1, there is no high certainty of the evidence on self-management interventions in IA, and most of the certainty of evidence is moderate or low. This is natural because self-management is a so-called ‘complex intervention’. In complex interventions, the efficacy of specific components is difficult to isolate.^65^ The majority of interventions included some sort of patient education, problem solving and cognitive–behavioural therapy which are to be expected in the context of self-management.

The interventions were delivered by a range of healthcare professionals including rheumatologists, nurses, psychologists, nutritionists, physiotherapists, occupational therapists, social workers and dieticians. Besides them, the multidisciplinary teams that delivered the interventions also included laypersons, pairs of lay leaders, counsellors and yoga teachers, although with a smaller participation.

No expert patients were involved in the delivery of education or interventions based on the published literature, which is in contrasts to what is being offered at least by some patient organisations. This observation was perhaps due to the setting of the studies (mainly hospitals (secondary care)), the year of the publication of the most long-standing studies which is still not sensitive to the growing patient research partners paradigm, and due to a pure research context of the study.

Surprisingly, only one study measured adherence as an outcome of patient education.^46^ Whereas several studies have examined adherence, only one RCT focused on the effect of self-management strategies (patient education) to improve it. This could be due to research bias or difficulties. The effect of patient education on adherence is positive, despite being based on a few patients and short time of follow-up.

Another issue that has not been the subject of this review, but that deserves attention, is the cost-effectiveness of the self-management interventions. Two of the excluded RCTs presented economic results and pointed out discrepancy in results. One,^66^ concluded that self-management programmes represent a cost-effective use of resources compared with usual care with a £20 000–£30 000/QALY gained and leads to lower healthcare costs and work absence. The other,^67^ suggested that although self-management improves the quality of life, it does so with a higher cost (Δ=€4211). This increased cost substantially reduced when medication costs were left out of the equation (Δ=€1863). Further economic studies are warranted to provide greater clarity on the subject.

In conclusion, several issues limit and make it difficult to state recommendations that can be made for the implementation of self-management interventions in IA. Well-structured self-management programmes are lacking or are poorly reported,^68^ and this is probably due to the articles’ word-count constraints. On the other hand, self-management behaviours are influenced by sociodemographic variables, health status and disease.^69^ This may lead to some components not having the same applicability between different contexts or countries. The multiform way of offering these interventions also makes it difficult to analyse their individual effectiveness because most of them are centred in hospitals, which is distorted by the very concept of self-management interventions. Professionals should look out for ‘new ways’ that are more adjusted and closer to the patient needs, such as internet programmes, which are proven to be effective in improving health status measures at 1 year.^62 67^ At last, there are even challenges in better defining which outcomes should be measured in self-management interventions.^70^ In the future, a formal outcomes core set should be established in self-management interventions.

## Data Availability

Data sharing not applicable as no datasets generated and/or analysed for this study. All data relevant to the study are included in the article or uploaded as supplementary information. Data sharing not applicable as no datasets generated and/or analysed for this study. All data relevant to the study are included in the article or uploaded as supplementary information.
