# Supplementary material for: Effectiveness of self-management interventions in inflammatory arthritis: a systematic review informing the 2021 EULAR recommendations for the implementation of self-management strategies in patients with inflammatory arthritis
Source: RMD Open. 2021 May 28;7(2):e001647. doi: 10.1136/rmdopen-2021-001647 (PMC8166594; doi:10.1136/rmdopen-2021-001647)
Supplement: Supplementary data [file rmdopen-2021-001647supp001.pdf]

Online supplementary material S1: Search strategies

PubMed: Searched on 20th January 2020

ID	Search Terms	Results
#1	Search (((((((((rheumat*[Title/Abstract]) OR reumat*[Title/Abstract]) OR arthrit*[Title/Abstract]) OR artrit*[Title/Abstract]) OR "arthritis, rheumatoid"[MeSH Terms]) OR psoriatic*[Title/Abstract]) OR arthritis, psoriatic[MeSH Terms]) OR spondylitis*[Title/Abstract]) OR spondyloarthropat*[Title/Abstract]) OR spondyloarthritis[Title/Abstract]) OR spondylitis, ankylosing[MeSH Terms]	295,303
#2	Search (((((((((((((((((((((((((((self-management[MeSH Terms]) OR self care[MeSH Terms]) OR patient education as topic[MeSH Terms]) OR self-help groups[MeSH Terms]) OR self efficacy[MeSH Terms]) OR behavior therapy[MeSH Terms]) OR counselling[MeSH Terms]) OR manag*[Title/Abstract]) OR monitor[Title/Abstract]) OR patient education[Title/Abstract]) OR helpline[Title/Abstract]) OR written information[Title/Abstract]) OR group-based course[Title/Abstract]) OR support group[Title/Abstract]) OR educat*[Title/Abstract]) OR program*[Title/Abstract]) OR tool*[Title/Abstract]) OR material*[Title/Abstract]) OR training[Title/Abstract]) OR app[Title/Abstract]) OR self-help[Title/Abstract]) OR support[Title/Abstract]) OR peer[Title/Abstract]) OR group*[Title/Abstract]) OR self-efficacy[Title/Abstract]) OR behav*[Title/Abstract]) OR coping[Title/Abstract]) OR skill*[Title/Abstract]) OR adaptive therapy[Title/Abstract]) OR relaxation[Title/Abstract]	8,619,378
#3	Search ((((((((((((((((((treatment outcome[MeSH Terms]) OR problem solving[MeSH Terms]) OR asthenia[MeSH Terms]) OR adaptation, psychological[MeSH Terms]) OR efficac*[Title/Abstract]) OR effective*[Title/Abstract]) OR implement*[Title/Abstract]) OR success[Title/Abstract]) OR patient-related outcomes[Title/Abstract]) OR pain[Title/Abstract]) OR functional disability[Title/Abstract]) OR fatigue[Title/Abstract]) OR tired*[Title/Abstract]) OR wear*[Title/Abstract]) OR exhaust*[Title/Abstract]) OR emotional well-being[Title/Abstract]) OR sleep*[Title/Abstract]) OR coping[Title/Abstract]) OR physical well-being[Title/Abstract]) OR asthen*[Title/Abstract]	4,533,513
#4	Search ((((((((((((rheumat*[Title/Abstract]) OR reumat*[Title/Abstract]) OR arthrit*[Title/Abstract]) OR artrit*[Title/Abstract]) OR "arthritis, rheumatoid"[MeSH Terms]) OR psoriatic*[Title/Abstract]) OR arthritis, psoriatic[MeSH Terms]) OR spondylitis*[Title/Abstract]) OR spondyloarthropat*[Title/Abstract]) OR spondyloarthritis[Title/Abstract]) OR spondylitis, ankylosing[MeSH Terms])) AND ((((((((((((((((((((((((((((self-management[MeSH Terms]) OR self care[MeSH Terms]) OR patient education as topic[MeSH Terms]) OR self-help groups[MeSH Terms]) OR self efficacy[MeSH Terms]) OR behavior therapy[MeSH Terms]) OR counselling[MeSH Terms]) OR manag*[Title/Abstract]) OR monitor[Title/Abstract]) OR patient education[Title/Abstract]) OR helpline[Title/Abstract]) OR written information[Title/Abstract]) OR group-based course[Title/Abstract]) OR support group[Title/Abstract]) OR educat*[Title/Abstract]) OR program*[Title/Abstract]) OR tool*[Title/Abstract]) OR material*[Title/Abstract]) OR training[Title/Abstract]) OR app[Title/Abstract]) OR self-help[Title/Abstract]) OR support[Title/Abstract]) OR peer[Title/Abstract]) OR group*[Title/Abstract]) OR self-efficacy[Title/Abstract]) OR	31,845

	behav*[Title/Abstract]) OR coping[Title/Abstract]) OR skill*[Title/Abstract]) OR adaptive therapy[Title/Abstract]) OR relaxation[Title/Abstract])) AND ((((((((((((((((((((treatment outcome[MeSH Terms]) OR problem solving[MeSH Terms]) OR asthenia[MeSH Terms]) OR adaptation, psychological[MeSH Terms]) OR efficac*[Title/Abstract]) OR effective*[Title/Abstract]) OR implement*[Title/Abstract]) OR success[Title/Abstract]) OR patient-related outcomes[Title/Abstract]) OR pain[Title/Abstract]) OR functional disability[Title/Abstract]) OR fatigue[Title/Abstract]) OR tired*[Title/Abstract]) OR wear*[Title/Abstract]) OR exhaust*[Title/Abstract]) OR emotional well-being[Title/Abstract]) OR sleep*[Title/Abstract]) OR coping[Title/Abstract]) OR physical well-being[Title/Abstract]) OR asthen*[Title/Abstract])	
#5	Search (randomized controlled trial[pt] OR controlled clinical trial[pt] OR randomized[tiab] OR placebo[tiab] OR drug therapy[sh] OR randomly[tiab] OR trial[tiab] OR groups[tiab] NOT (animals [mh] NOT humans [mh]))	4,053,361
#6	Search ((((((((((((((((rheumat*[Title/Abstract]) OR reumat*[Title/Abstract]) OR arthrit*[Title/Abstract]) OR artrit*[Title/Abstract]) OR "arthritis, rheumatoid"[MeSH Terms]) OR psoriatic*[Title/Abstract]) OR arthritis, psoriatic[MeSH Terms]) OR spondylitis*[Title/Abstract]) OR spondyloarthropat*[Title/Abstract]) OR spondyloarthritis[Title/Abstract]) OR spondylitis, ankylosing[MeSH Terms])) AND ((((((((((((((((((((((((((((((self-management[MeSH Terms]) OR self care[MeSH Terms]) OR patient education as topic[MeSH Terms]) OR self-help groups[MeSH Terms]) OR self efficacy[MeSH Terms]) OR behavior therapy[MeSH Terms]) OR counselling[MeSH Terms]) OR manag*[Title/Abstract]) OR monitor[Title/Abstract]) OR patient education[Title/Abstract]) OR helpline[Title/Abstract]) OR written information[Title/Abstract]) OR group-based course[Title/Abstract]) OR support group[Title/Abstract]) OR educat*[Title/Abstract]) OR program*[Title/Abstract]) OR tool*[Title/Abstract]) OR material*[Title/Abstract]) OR training[Title/Abstract]) OR app[Title/Abstract]) OR self-help[Title/Abstract]) OR support[Title/Abstract]) OR peer[Title/Abstract]) OR group*[Title/Abstract]) OR self-efficacy[Title/Abstract]) OR behav*[Title/Abstract]) OR coping[Title/Abstract]) OR skill*[Title/Abstract]) OR adaptive therapy[Title/Abstract]) OR relaxation[Title/Abstract])) AND ((((((((((((((((((((treatment outcome[MeSH Terms]) OR problem solving[MeSH Terms]) OR asthenia[MeSH Terms]) OR adaptation, psychological[MeSH Terms]) OR efficac*[Title/Abstract]) OR effective*[Title/Abstract]) OR implement*[Title/Abstract]) OR success[Title/Abstract]) OR patient-related outcomes[Title/Abstract]) OR pain[Title/Abstract]) OR functional disability[Title/Abstract]) OR fatigue[Title/Abstract]) OR tired*[Title/Abstract]) OR wear*[Title/Abstract]) OR exhaust*[Title/Abstract]) OR emotional well-being[Title/Abstract]) OR sleep*[Title/Abstract]) OR coping[Title/Abstract]) OR physical well-being[Title/Abstract]) OR asthen*[Title/Abstract]))) AND ((randomized controlled trial[pt] OR controlled clinical trial[pt] OR randomized[tiab] OR placebo[tiab] OR drug therapy[sh] OR randomly[tiab] OR trial[tiab] OR groups[tiab] NOT (animals [mh] NOT humans [mh])))) AND systematic [sb]	715

Embase: Searched on 24th January 2020

ID	Search Terms	Results
#1	((('rheumat*':ab,ti OR 'reumat*':ab,ti OR 'arthrit*':ab,ti OR 'artrit*':ab,ti OR 'psoriatic*':ab,ti OR 'spondylitis*':ab,ti OR 'spondyloarthropat*':ab,ti OR 'spondyloarthritis':ab,ti) AND 'rheumatoid arthritis'/exp AND 'psoriatic arthritis'/exp AND 'ankylosing spondylitis'/exp AND 'manag*':ab,ti OR 'monitor':ab,ti OR 'patient education':ab,ti OR 'helpline':ab,ti OR 'written information':ab,ti OR 'group-based course':ab,ti OR 'support group':ab,ti OR 'educat*':ab,ti OR 'program*':ab,ti OR 'tool*':ab,ti OR 'material*':ab,ti OR 'training':ab,ti OR 'app':ab,ti OR 'self-help':ab,ti OR 'support':ab,ti OR 'peer':ab,ti OR 'group*':ab,ti OR 'self-efficacy':ab,ti OR 'behav*':ab,ti OR 'coping':ab,ti OR 'skill*':ab,ti OR 'adaptive therapy':ab,ti OR 'relaxation':ab,ti) AND 'self care'/exp AND 'patient education'/exp AND 'self help'/exp AND 'self concept'/exp AND 'behavior therapy'/exp AND 'counseling'/exp AND 'efficac*':ab,ti OR 'effective*':ab,ti OR 'implement*':ab,ti OR 'success':ab,ti OR 'patient-related outcomes':ab,ti OR 'pain':ab,ti OR 'functional disability':ab,ti OR 'fatigue':ab,ti OR 'tired*':ab,ti OR 'wear*':ab,ti OR 'exhaust*':ab,ti OR 'emotional well-being':ab,ti OR 'sleep*':ab,ti OR 'coping':ab,ti OR 'physical well-being':ab,ti OR 'asthen*':ab,ti) AND 'treatment outcome'/exp AND 'problem solving'/exp OR 'coping behavior'/exp	63,245
#2	'crossover procedure':de OR 'double-blind procedure':de OR 'randomized controlled trial':de OR 'single-blind procedure':de OR random*:de,ab,ti OR factorial*:de,ab,ti OR crossover*:de,ab,ti OR ((cross NEXT/1 over*):de,ab,ti) OR placebo*:de,ab,ti OR ((doubl* NEAR/1 blind*):de,ab,ti) OR ((singl* NEAR/1 blind*):de,ab,ti) OR assign*:de,ab,ti OR allocat*:de,ab,ti OR volunteer*:de,ab,ti	2,549,613
#3	'systematic review'/syn OR 'meta analysis'/syn	432,662
#4	#1 AND #2 AND #3	718

Cochrane Library: Searched on 20th January 2020

ID	Search Terms	Results
#1	(rheumat* OR reumat* OR arthrit* OR artrit* OR psoriatic* OR spondylitis* OR spondyloarthropat* OR spondyloarthritis):ti,ab,kw in Cochrane Reviews, Trials	28,562
#2	MeSH descriptor: [Arthritis, Rheumatoid] explode all trees	5,573
#3	MeSH descriptor: [Arthritis, Psoriatic] explode all trees	364
#4	MeSH descriptor: [Spondylitis, Ankylosing] explode all trees	617
#5	#1 AND #2 OR #3 OR #4 in Cochrane Reviews, Trials	6,195
#6	(manag* OR monitor OR patient education OR helpline OR written information OR group-based course OR support group OR educat* OR program* OR tool* OR material* OR training OR app OR self-help OR support OR peer OR group* OR self-efficacy OR	916,217

	behav* OR coping OR skill* OR adaptive therapy OR relaxation):ti,ab,kw in Cochrane Reviews, Trials	
#7	MeSH descriptor: [Self-Management] explode all trees	263
#8	MeSH descriptor: [Self Care] explode all trees	5,418
#9	MeSH descriptor: [Patient Education as Topic] explode all trees	8,521
#10	MeSH descriptor: [Self-Help Groups] explode all trees	755
#11	MeSH descriptor: [Self Efficacy] explode all trees	2,933
#12	MeSH descriptor: [Behavior Therapy] explode all trees	15,643
#13	MeSH descriptor: [Counseling] explode all trees	5,121
#14	#6 AND #7 OR #8 OR #9 OR #10 OR #11 OR #12 OR #13 in Cochrane Reviews, Trials	32,645
#15	(efficac* OR effective* OR implement* OR success OR patient-related outcomes OR pain OR functional disability OR fatigue OR tired* OR wear* OR exhaust* OR emotional well-being OR sleep* OR coping OR physical well-being OR asthen*):ti,ab,kw in Cochrane Reviews, Trials	688,974
#16	MeSH descriptor: [Treatment Outcome] explode all trees	133,928
#17	MeSH descriptor: [Problem Solving] explode all trees	1,510
#18	MeSH descriptor: [Asthenia] explode all trees	74
#19	MeSH descriptor: [Adaptation, Psychological] explode all trees	5,209
#20	#15 AND #16 OR #17 OR #18 OR #19 in Cochrane Reviews, Trials	89,928
#21	#5 AND #14 AND #20 in Cochrane Reviews, Trials	72

CINAHL Complete: Searched on 23rd January 2020

ID	Search Terms	Results
S1	AB MH Randomized controlled trials OR MH double-blind studies OR MH single-blind studies OR MH random assignment OR MH pretest-posttest design OR MH cluster sample OR TI (comparative or comparison) AND AB (randomly) OR TI (randomized or randomised) NOT MH (rats)	233,593
S2	(TI (rheumat* OR reumat* OR arthrit* OR artrit* OR psoriatic* OR spondylitis* OR spondyloarthropat* OR spondyloarthritis OR (MH "arthritis, rheumatoid") OR (MH “arthritis, psoriatic”) OR (MH “spondylitis, ankylosing”)) AND TI (manag* OR monitor OR patient education OR helpline OR written information OR group-based course OR support group OR educat* OR program* OR tool* OR material* OR training OR app OR self-help	8,540

	OR support OR peer OR group* OR self-efficacy OR behav* OR coping OR skill* OR adaptive therapy OR relaxation OR (MH “self-management”) OR (MH “self care”) OR (MH “patient education as topic”) OR (MH “self-help groups”) OR (MH “self efficacy”) OR (MH “behavior therapy”) OR (MH “counselling”)) AND TI (efficac* OR effective* OR implement* OR success OR patient-related outcomes OR pain OR functional disability OR fatigue OR tired* OR wear* OR exhaust* OR emotional well-being OR sleep* OR coping OR physical well-being OR asthen* OR (MH “treatment outcome”) OR (MH “problem solving”) OR (MH “astenia”) OR (MH “adaptation, psychological”))) OR (AB (rheumat* OR reumat* OR arthrit* OR artrit* OR psoriatic* OR spondylitis* OR spondyloarthropat* OR spondyloarthritis OR (MH "arthritis, rheumatoid") OR (MH “arthritis, psoriatic”) OR (MH “spondylitis, ankylosing”)) AND AB (manag* OR monitor OR patient education OR helpline OR written information OR group-based course OR support group OR educat* OR program* OR tool* OR material* OR training OR app OR self-help OR support OR peer OR group* OR self-efficacy OR behav* OR coping OR skill* OR adaptive therapy OR relaxation OR (MH “self-management”) OR (MH “self care”) OR (MH “patient education as topic”) OR (MH “self-help groups”) OR (MH “self efficacy”) OR (MH “behavior therapy”) OR (MH “counselling”)) AND AB (efficac* OR effective* OR implement* OR success OR patient-related outcomes OR pain OR functional disability OR fatigue OR tired* OR wear* OR exhaust* OR emotional well-being OR sleep* OR coping OR physical well-being OR asthen* OR (MH “treatment outcome”) OR (MH “problem solving”) OR (MH “astenia”) OR (MH “adaptation, psychological”)))	
S3	Meta analysis/ OR Meta analys$.tw. OR Metaanaly$.tw. OR exp Literature review/ OR (systematic adj (review or overview)).tw. NOT Commentary.pt. OR Letter.pt. OR Editorial.pt. OR Animals/	283,919
S4	(S1 AND S2) AND S3)	67

PEDro: Searched on 23rd January 2020

**Search Terms**	**Results**
Abstract & Title: self-management AND inflammatory arthritis	5
