# Supplementary material for: Effectiveness of self-management interventions in inflammatory arthritis: a systematic review informing the 2021 EULAR recommendations for the implementation of self-management strategies in patients with inflammatory arthritis
Source: RMD Open. 2021 May 28;7(2):e001647. doi: 10.1136/rmdopen-2021-001647 (PMC8166594; doi:10.1136/rmdopen-2021-001647)
Supplement: Supplementary data [file rmdopen-2021-001647supp002.pdf]

Online supplementary material S2: Excluded articles

Reference	Participants	Interventions	Outcomes	Type of study
Bal MI, Sattoe JNT, Roelofs PDDM, Bal R, van Staa A, Miedema HS. Exploring effectiveness and effective components of self-management interventions for young people with chronic physical conditions: A systematic review. Patient Education and Counseling. 2016;99:1293-309.	NO	YES	YES	YES
Banerjee A, Hendrick P, Bhattacharjee P, Blake H. A systematic review of outcome measures utilised to assess self-management in clinical trials in patients with chronic pain. Patient Education and Counseling. 2018;101:767-78.	DOUBT	DOUBT	NO	YES
Chaleshgar Kordasiabi M, Akhlaghi M, Baghianimoghadam MH, Morowatisharifabad MA, Askarishahi M, Enjezab B, et al. Self Management Behaviors in Rheumatoid Arthritis Patients and Associated Factors in Tehran 2013. Global journal of health science. 2015;8:156-67.	YES	NO	NO	NO
de Thurah A, Stengaard-Pedersen K, Axelsen M, Fredberg U, Schougaard LMV, Hjollund NHI, et al. Tele-Health Followup Strategy for Tight Control of Disease Activity in Rheumatoid Arthritis: Results of a Randomized Controlled Trial. Arthritis Care Res (Hoboken). 2018;70:353-60.	YES	NO	YES	YES
Eisele A, Schagg D, Krämer LV, Bengel J, Göhner W. Behaviour change techniques applied in interventions to enhance physical activity adherence in patients with chronic musculoskeletal conditions: A systematic review and meta-analysis. Patient Education and Counseling. 2019;102:25-36.	NO	YES	YES	YES
Ferwerda M, van Beugen S, van Middendorp H, Visser H, Vonkeman H, Creemers M, et al. Tailored, Therapist-Guided Internet-Based Cognitive Behavioral Therapy Compared to Care as Usual for Patients With Rheumatoid Arthritis: Economic Evaluation of a Randomized Controlled Trial. J Med Internet Res. 2018;20:e260.	YES	YES	NO	YES

Karimipour H, Sayadi N, Shariati A, Haghighi MH, Yaghoubi R. The Effects of a Self-Care Program on Promoting Self-Care Behaviors in Patients with Psoriasis. Jundishapur J Chronic Dis Care. 2017;6:e17378.	NO	YES	YES	YES
Keefe FJ, Lefebvre JC, Kerns RD, Rosenberg R, Beaupre P, Prochaska J, et al. Understanding the adoption of arthritis self-management: stages of change profiles among arthritis patients. Pain. 2000;87:303-13.	DOUBT	DOUBT	DOUBT	NO
Keogh A, Tully MA, Matthews J, Hurley DA. A review of behaviour change theories and techniques used in group based self-management programmes for chronic low back pain and arthritis. Manual Therapy. 2015;20:727-35.	NO	DOUBT	DOUBT	YES
Lambert S, Beatty L, McElduff P, Levesque J, Lawsin C, Turner J, et al. Are self-administered psychosocial interventions as efficacious as guided interventions in the management of chronic health conditions? Asia-Pacific Journal of Clinical Oncology. 2015;11:151.	NO	YES	YES	YES
Lambert S, Beatty L, McElduff P, Levesque J, Lawsin C, Turner J, et al. Evaluations of written self-administered psychosocial interventions to improve psychosocial and physical outcomes among adults with chronic health conditions: A meta-analysis. Psycho-Oncology. 2015;24:73.	NO	YES	YES	YES
Lambert SD, Beatty L, McElduff P, Levesque JV, Lawsin C, Jacobsen P, et al. A systematic review and meta-analysis of written self-administered psychosocial interventions among adults with a physical illness. Patient Education and Counseling. 2017;100:2200-17.	NO	YES	YES	YES
Larsen MH, Hagen KB, Krogstad AL, Aas E, Wahl AK. Limited evidence of the effects of patient education and self-management interventions in psoriasis patients: A systematic review. Patient Education and Counseling. 2014;94:158-69.	NO	YES	YES	YES
Lorig K, Ritter PL, Plant K. A disease-specific self-help program compared with a generalized chronic disease self-help program for arthritis patients. Arthritis Rheum. 2005;53:950-7.	NO	YES	YES	YES

Lorig KR, Ritter PL, Laurent DD, Plant K. The internet-based arthritis self-management program: a one-year randomized trial for patients with arthritis or fibromyalgia. Arthritis Rheum. 2008;59:1009-17.	NO	YES	YES	YES
Manning VL, Kaambwa B, Ratcliffe J, Scott DL, Choy E, Hurley MV, et al. Economic evaluation of a brief education, self-management and upper limb exercise training in people with rheumatoid arthritis (EXTRA) programme: a trial-based analysis. Rheumatology (Oxford). 2015;54:302-9.	YES	YES	NO	YES
Rasmussen GS, Kragballe K, Maindal HT, Lomborg K. Experience of Being Young With Psoriasis: Self-Management Support Needs. Qualitative Health Research. 2017;28:73-86.	NO	DOUBT	DOUBT	DOUBT
Rasmussen GS, Maindal HT, Lomborg K. Self-management in daily life with psoriasis: an integrative review of patient needs for structured education. Nursing research and practice. 2012;2012:890860-.	NO	DOUBT	DOUBT	DOUBT
Rees S, Williams A. Promoting and supporting self-management for adults living in the community with physical chronic illness: A systematic review of the effectiveness and meaningfulness of the patient-practitioner encounter. JBI Libr Syst Rev. 2009;7:492-582.	NO	DOUBT	DOUBT	YES
Rhee SH, Parker JC, Smarr KL, Petroski GF, Johnson JC, Hewett JE, et al. Stress management in rheumatoid arthritis: what is the underlying mechanism? Arthritis Care Res. 2000;13:435-42.	YES	YES	NO	NO
Shigaki CL, Smarr KL, Gong Y, Donovan-Hanson K, Siva C, Johnson RA, et al. Social interactions in an online self-management program for rheumatoid arthritis. Chronic Illn. 2008;4:239-46.	YES	YES	DOUBT	NO
Solomon DH, Warsi A, Brown-Stevenson T, Farrell M, Gauthier S, Mikels D, et al. Does self-management education benefit all populations with arthritis? A randomized controlled trial in a primary care physician network. J Rheumatol. 2002;29:362-8.	NO	YES	YES	YES

Spencer S, Martindale JH, MacPhie E, Montgomery P. Self management programme for ankylosing spondylitis. Cochrane Database of Systematic Reviews. 2015.	YES	YES	YES	NO
Vermaak V, Briffa NK, Langlands B, Inderjeeth C, McQuade J. Evaluation of a disease specific rheumatoid arthritis self-management education program, a single group repeated measures study. BMC Musculoskeletal Disorders. 2015;16:214.	YES	YES	YES	NO
Niedermann K, de Bie RA, Kubli R, Ciurea A, Steurer-Stey C, Villiger PM, et al. Effectiveness of individual resource-oriented joint protection education in people with rheumatoid arthritis. A randomized controlled trial. Patient Educ Couns. 2011;82:42-8.	YES	YES	YES	NO

Yes: the study meets the inclusion criteria; No: the study does not meet the inclusion criteria; Doubt: the decision was not consensual between the two reviewers and the third was included as a tiebreaker criterion.
