# Supplementary material for: Effectiveness of self-management interventions in inflammatory arthritis: a systematic review informing the 2021 EULAR recommendations for the implementation of self-management strategies in patients with inflammatory arthritis
Source: RMD Open. 2021 May 28;7(2):e001647. doi: 10.1136/rmdopen-2021-001647 (PMC8166594; doi:10.1136/rmdopen-2021-001647)
Supplement: Supplementary data [file rmdopen-2021-001647supp003.pdf]

Online supplementary material S3: Assessment of risk of bias

Risk of bias summary graph for included clinical trials: review authors' judgements about each risk of bias item presented as percentages across all included studies using the Cochrane RoB tool.

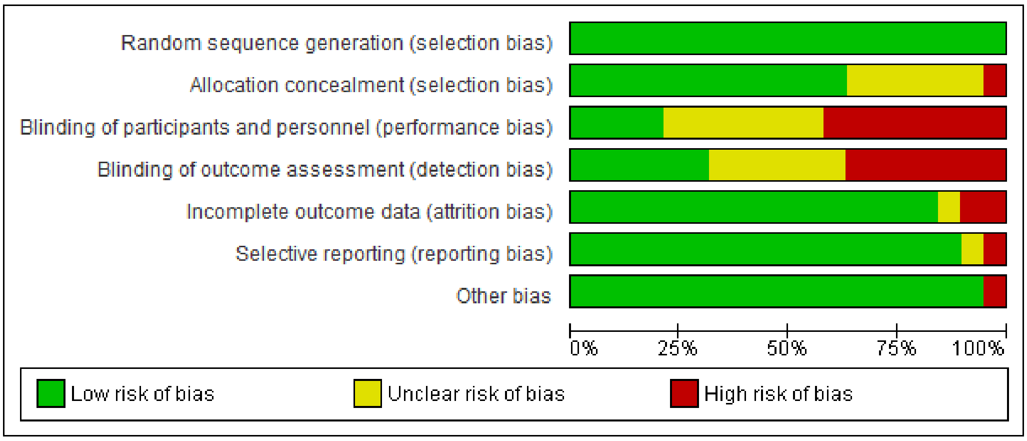

Risk of bias of included clinical trials: Review authors' judgements about each risk of bias item of the Cochrane's RoB tool for each study.

	Random sequence generation (selection bias)	Allocation concealment (selection bias)	Blinding of participants and personnel (performance bias)	Blinding of outcome assessment (detection bias)	Incomplete outcome data (attrition bias)	Selective reporting (reporting bias)	Other bias
Barlow et al., 1998	+	?	−	?	+	+	+
Barsky et al., 2010	+	+	+	+	+	+	+
Basler et al., 1991	+	?	−	−	?	+	+
El Miedany et al., 2012	+	?	?	?	+	+	+
Evers et al., 2002	+	?	−	−	+	+	+
Freeman et al., 2002	+	+	?	+	+	+	+
Giraudet-Le Quintrec 2007	+	+	?	?	+	+	+
Hammond et al., 2008	+	+	−	−	+	+	+
Hewlett et al., 2011	+	+	−	−	+	+	+
Hill et al., 2001	+	+	+	+	+	+	+
Knittle et al., 2013	+	+	+	?	+	+	+
Lumley et al. 2014	+	?	?	−	+	?	+
Manning et al., 2014	+	+	?	?	+	+	+
Niedermann et al., 2011	+	+	+	+	+	+	+
Seneca et al., 2015	+	+	?	+	+	+	+
Shearn et al., 1985	+	−	−	−	−	−	−
Shigaki et al., 2013	+	?	−	+	+	+	+
van Lankveld et al., 2004	+	+	−	−	−	+	+
Zuidema et al., 2019	+	+	?	?	+	+	+

Risk of bias summary for systematic reviews using the AMSTAR2 tool: Review authors' judgements about each risk of bias item for each included study.

Study	Q1	Q2	Q3	Q4	Q5	Q6	Q7	Q8	Q9	Q10	Q11	Q12	Q13	Q14	Q15	Q16	Quality
Astin, 2002	Y	N	Y	PY	N	Y	N	Y	Y	N	Y	Y	Y	Y	Y	Y	Moderate
Carandang, 2016	Y	N	N	PY	N	Y	N	PY	Y	N	NM	NM	Y	N	NM	Y	Moderate
Cramer, 2013	Y	N	N	PY	Y	Y	N	Y	Y	N	NM	NM	Y	N	NM	Y	Moderate
Cramp, 2013	Y	Y	Y	Y	Y	Y	Y	Y	Y	Y	Y	Y	Y	Y	Y	Y	High
Dagfinrud, 2008	Y	Y	Y	Y	Y	Y	Y	Y	Y	Y	NM	NM	Y	Y	NM	Y	Moderate
DiRenzo, 2018	Y	N	N	Y	Y	Y	N	Y	Y	N	Y	Y	Y	Y	N	Y	Moderate
Dissanayake, 2010	Y	N	N	PY	Y	Y	N	Y	Y	N	NM	NM	Y	N	NM	N	Moderate
Du, 2011	Y	N	N	PY	Y	Y	N	Y	Y	N	Y	Y	Y	Y	N	Y	Moderate
Knittle, 2010	Y	N	N	PY	Y	Y	N	Y	Y	N	Y	Y	Y	Y	Y	Y	Moderate
Lopes, 2016	Y	N	N	N	N	Y	N	PY	PY	N	NM	NM	N	N	NM	N	Low
Mudano, 2019	Y	Y	Y	Y	Y	Y	Y	Y	Y	Y	Y	Y	Y	Y	Y	Y	High
Pécourneau, 2018	Y	N	N	PY	N	N	N	Y	Y	N	Y	Y	Y	N	N	Y	Moderate
Riemsma, 2003	Y	Y	Y	Y	Y	Y	Y	Y	Y	N	Y	Y	Y	Y	Y	Y	High
% YES	100	31	38.5	38.5	69.2	92.3	31	84.6	92.3	23.1	61.5	61.5	92.3	61.5	38.5	84.6	

Y: Yes; N: No; PY: Partial Yes; NM: No Meta-analysis conducted.

Q1 = Did the research questions and inclusion criteria for the review include the components of PICO?; Q2 = Did the report of the review contain an explicit statement that the review methods were established prior to the conduct of the review and did the report justify any significant deviations from the protocol?; Q3 = Did the review authors explain their selection of the study designs for inclusion in the review?; Q4 = Did the review authors use a comprehensive literature search strategy?; Q5 = Did the review authors perform study selection in duplicate?; Q6 = Did the review authors perform data extraction in duplicate?; Q7 = Did the review authors provide a list of excluded studies and justify the exclusions?; Q8 = Did the review authors describe the included studies in adequate detail?; Q9 = Did the review authors use a satisfactory technique for assessing the risk of bias (RoB) in individual studies that were included in the review?; Q10 = Did the review authors report on the sources of funding for the studies included in the review?; Q11 = If meta-analysis was performed did the review authors use appropriate methods for statistical combination of results?; Q12 = If meta-analysis was performed, did the review authors assess the potential impact of RoB in individual studies on the results of the meta-analysis or other evidence synthesis? Q13 = Did the review authors account for RoB in individual studies when interpreting/ discussing the results of the review?; Q14 = Did the review authors provide a satisfactory explanation for, and discussion of, any heterogeneity observed in the results of the review?; Q15 = If they performed quantitative synthesis did the review authors carry out an adequate investigation of publication bias (small study bias) and discuss its likely impact on the results of the review?; Q16 = Did the review authors report any potential sources of conflict of interest, including any funding they received for conducting the review?
