# Supplementary material for: Effectiveness of self-management interventions in inflammatory arthritis: a systematic review informing the 2021 EULAR recommendations for the implementation of self-management strategies in patients with inflammatory arthritis
Source: RMD Open. 2021 May 28;7(2):e001647. doi: 10.1136/rmdopen-2021-001647 (PMC8166594; doi:10.1136/rmdopen-2021-001647)
Supplement: Supplementary data [file rmdopen-2021-001647supp004.pdf]

Online supplementary material S4: Details of included studies

Contents

Systematic reviews 2
Astin et al., 2002 2
Cramp et al., 2013 3
Carandang et al., 2016 4
Cramer et al., 2013 5
Dagfinrud et al., 2008 6
DiRenzo et al., 2018 7
Dissanayake et al., 2010 8
Du et al., 2011 9
Knittle et al., 2010 10
Lopes et al., 2016 11
Mudano et al., 2019 12
Pécourneau et al. 2018 13
Riemsma et al., 2003 14

Randomised controlled trials 15
Barlow et al., 1998 & Barlow et al., 1997 15
Barsky et al., 2010 16
Basler et al., 1991 17
El Miedany et al., 2012 18
Evers et al., 2002 19
Freeman et al., 2002 20
Giraudet-Le Quintrec et al., 2007 21
Hammond et al., 2008 22
Hewlett et al., 2011 23
Hill et al., 2001 24
Knittle et al., 2013 25
Lumley et al., 2014 26
Manning et al., 2014 27
Niedermann et al., 2011 & Niedermann et al., 2012 28
Seneca et al., 2015 29
Shearn et al., 1985 30
Shigaki et al., 2013 31
van Lankveld et al., 2004 32
Zuidema et al 2019 33

Abbreviations

AS	Ankylosing spondylitis
CBT	Cognitive behavioural therapy
FM	Fibromyalgia
g	Hedges' g effect size
NA	Not applicable
NS	Not Significant
OA	Osteoarthritis
PE	Patient education
PGA	Patient global Assessment
PROMs	Patient reported outcome measures
PsA	Psoriatic arthritis
RA	Rheumatoid arthritis
RCT	Randomised controlled trial
RPD	Relative percentage difference
SMD	Standardised mean difference
WMD	Weighted mean difference

Systematic reviews

Astin et al., 2002		
Databases searched and language limits	MEDLINE, PsychLit, EMBASE, CAM-PAIN, Science Citation Index, and Cochrane Library<br>English language.	
Range of included studies	1983-2001	
Number, type of studies included and countries of origin	25 RCTs<br>Not stated	
Instruments used for bias appraisal	Jadad scale (10-point quality rating scale)	
Bias appraisal rating	Mean score 5.84, range 3–9.	
Participants characteristics (number, age, disease criteria, details)	RA patients (n= 1,676)<br>Disease duration 10.6 years	
Intervention(s)	Psychological/psychosocial component beyond simply providing information (e.g., patient education) about the disease, characterized as multimodal, cognitive–behavioral interventions which involve the combination of relaxation, biofeedback, imagery, stress management, or the teaching of cognitive coping skills.	
Intervention(s) characteristics	Group based (2 studies) and individual (3 studies)<br>Other characteristics not stated	
Professional that promoted the interventions	Not stated	
Intervention(s) setting	Not stated	
Control	Usual medical care, Social support, Support group, Symptom monitoring wait list control, Occupational therapy, Attention control (education), Non-intervention control	
Outcomes of interest (types and measuring instruments)	Pain, functional disability (HAQ, AIMS, …), psychological status (depression; measured by AIMS, BDI, CES-D), coping, self-efficacy (or helplessness; measured by ASES, AHI), and tender joints.	
Methods of analysis	Meta-analysis + Narrative synthesis	
Heterogeneity (I^2)	*Q*-test (NS).	
Effect size	Pain (g=0.22)<br>Functional disability (g=0.27)<br>Tender joints (g=0.15)<br>Psychological status (g=0.15)<br>Coping (g=0.46)<br>Self-efficacy (g=0.35).	At follow-up:<br>Pain (g=0.06)<br>Functional disability (g=0.12)<br>Tender joints (g=0.30)<br>Psychological status (g=0.33)<br>Coping (g=0.52)<br>Self-efficacy (g=0.20).
95% Confidence intervals	Pain 0.07 to 0.37<br>Functional disability 0.12 to 0.42<br>Tender joints -0.09 to -0.39<br>Psychological status -0.01 to -0.31<br>Coping 0.09 to 0.83<br>Self-efficacy 0.11 to 0.59.	At follow-up:<br>Pain -0.17 to 0.29<br>Functional disability -0.09 to -0.33<br>Tender joints 0.04 to 0.56<br>Psychological status -0.07 to -0.59<br>Coping -0.07 to 1.11<br>Self-efficacy -0.08 to -0.48.
P value	Pain 0.003<br>Functional disability <0.005<br>Tender joints NS<br>Psychological status 0.03<br>Coping 0.007<br>Self-efficacy 0.02.	At follow-up:<br>Pain NS<br>Functional disability NS<br>Tender joints 0.005<br>Psychological status 0.01<br>Coping 0.04 and Self-efficacy NS.
Follow-up	Length of the interventions ranging from 3 days to 9 months with a mean of 9.8 weeks.<br>Follow-up time periods ranging from 2 to 18 months (mean of 8.6).	
Conclusions	Psychological interventions may be important adjunctive therapies in the medical management of RA.	

Cramp et al., 2013	
Databases searched and language limits	Cochrane Central Register of Controlled Trials, MEDLINE, EMBASE, AMED, CINAHL, PsycINFO, Social Science Citation Index, Web of Science, Dissertation Abstracts International, Current Controlled Trials Register (USA), The National Research Register (NRR) Archive (UK), The UKCRN Portfolio Database (UK)<br>English (other languages are not stated)
Range of included studies	1985 - 2012
Number, type of studies included and countries of origin	24 RCTs<br>Countries not stated
Instruments used for bias appraisal	Cochrane Collaboration appraisal tools
Bias appraisal rating	Quality of the evidence ranged from moderate quality for physical activity interventions and Mediterranean diet to low quality for psychosocial interventions and all other interventions.
Participants characteristics (number, age, disease criteria, details)	RA patients (n=2882)<br>Age 18-70 years<br>ACR criteria
Intervention(s)	6 × Physical activity interventions (pool-based therapy, Yoga, strength training, stationary cycling, aerobic exercise, Tai Chi)<br>13 × Psychosocial interventions (expressive writing, cognitive skills training, 2 × cognitive behavioral therapy, mindfulness, lifestyle management, education incorporating energy conservation, 3 × education incorporating self-management, 3 × group education)<br>1 × Herbal medicine<br>1 × Omega-3 fatty acid supplementation<br>1 × Mediterranean diet<br>1 × Reflexology<br>1 × Health Tracker information
Intervention(s) characteristics	Frequency 2-3 times a daily<br>Duration 20 min-4.5 hours<br>Intensity moderate (majority unstated)
Professional that promoted the interventions	Yoga teachers, Physiotherapists, Occupational therapists, Clinical psychologists, Nurses, Dieticians
Intervention(s) setting	Class - at a fitness centre, Home, majority unstated
Control	Not stated
Outcomes of interest (types and measuring instruments)	Fatigue [VAS, SF-36, the Multidimensional Assessment of Fatigue (MAF), Profile of Mood States (POMS), FACIT-F, Checklist Individual Strength (CIS), and the perception of change in fatigue from baseline using a four-point Likert scale]<br>Pain [VAS or NRS, Likert scale, short-form McGill Pain Questionnaire, AIMS2, the Impact of Rheumatic Diseases on General Health and Lifestyle (IRGL), Pain Disability Index, the Bodily Pain subscale of the<br>SF-36, and the Manchester Foot Pain Disability Questionnaire]<br>Functional disability (HAQ, IRGL and the AIMS2)
Methods of analysis	Meta-analysis + Narrative synthesis
Heterogeneity (I^2)	Fatigue: Physical activity interventions 27% low<br>Fatigue: Psychosocial interventions 55% moderate
Effect size	Fatigue: Physical activity interventions (n=371) SMD= -0.36 small<br>Fatigue: Psychosocial interventions (n=1556) SMD= -0.24 small<br>For the remaining interventions/ outcomes meta-analysis was not possible and there was either no statistically significant difference between trial arms or findings were not reported.
95% Confidence intervals	Fatigue: Physical activity interventions -0.62 to -0.10<br>Fatigue: Psychosocial interventions -0.40 to -0.07
P value	Fatigue: Physical activity interventions 0.0066<br>Fatigue: Psychosocial interventions 0.0044
Follow-up	3 - 24 months
Conclusions	Physical activity and psychosocial interventions provide benefit in relation to self-reported fatigue in adults with rheumatoid arthritis. There is currently insufficient evidence of the effectiveness of other non-pharmacological interventions.

Carandang et al., 2016	
Databases searched and language limits	MEDLINE and CINAHL.<br>English language.
Range of included studies	January 2002 - June 2015
Number, type of studies included and countries of origin	22 RCTs<br>Countries not stated
Instruments used for bias appraisal	Cochrane Collaboration appraisal tools
Bias appraisal rating	12 studies have low risk of bias,<br>3 studies had high risk of bias,<br>7 studies had unknown risk of bias.
Participants characteristics (number, age, disease criteria, details)	RA patients (n=2600)<br>Age >18 years<br>ACR criteria
Intervention(s)	Educational Interventions, Joint protection and energy conservation, Disease education, Pain management, Range of motion and strengthening, Provision of orthoses, Physical agent modalities, Cognitive rehabilitation, Environmental adaptation, Provision of adaptive equipment, Maintaining activities
Intervention(s) characteristics	Not stated
Professional that promoted the interventions	Occupational therapists
Intervention(s) setting	Not stated
Control	Not stated
Outcomes of interest (types and measuring instruments)	Pain and Fatigue
Methods of analysis	Narrative synthesis
Heterogeneity (I^2)	NA
Effect size	NA
95% Confidence intervals	NA
P value	NA
Follow-up	NA
Conclusions	Interventions in which a combination of educational techniques is used may complement pharmacological therapies in the care of people with RA.

Cramer et al., 2013	
Databases searched and language limits	Medline/PubMed, Scopus, the Cochrane Library and IndMED<br>Language limits not stated
Range of included studies	Through 11 February 2013
Number, type of studies included and countries of origin	8 RCTs (only 2 in RA)<br>USA, Brazil and India.
Instruments used for bias appraisal	Cochrane Collaboration appraisal tools
Bias appraisal rating	Quality of the evidence ranged from low to high.<br>The two RCTs in RA had high risk of bias.
Participants characteristics (number, age, disease criteria, details)	RA patients (n=110)<br>Aged between 29 and 35 years<br>70% female
Intervention(s)	Yoga, including postures, cleansing practices, breathing techniques, meditation, lifestyle advice and relaxation
Intervention(s) characteristics	180-540 min/week
Professional that promoted the interventions	Not stated
Intervention(s) setting	Not stated
Control	Usual care
Outcomes of interest (types and measuring instruments)	Pain (SDPIS), Disability (PDI, HAQ-DI), quality of life (SF-36), distress (BSI)
Methods of analysis	Narrative synthesis
Heterogeneity (I2)	NA
Effect size	NA
95% Confidence intervals	NA
P value	NA
Follow-up	6-7 weeks
Conclusions	Weak recommendations can be made for the ancillary use of yoga in the management of FM syndrome, OA and RA.

Dagfinrud et al., 2008	
Databases searched and language limits	Cochrane Central Register of Controlled Trials (CENTRAL), MEDLINE, EMBASE, AMED, CINAHL and PEDro<br>No language restrictions
Range of included studies	Up to January 2007
Number, type of studies included and countries of origin	11 RCTs<br>Netherlands, Spain, UK, Turkey, Canada, Korea
Instruments used for bias appraisal	Cochrane Collaboration appraisal tools
Bias appraisal rating	Quality of the evidence ranged from low to moderate.
Participants characteristics (number, age, disease criteria, details)	Ankylosing Spondylitis patients (AS)(n=763)<br>Aged between 13-69 years<br>67-93% men<br>Modified New York criteria
Intervention(s)	Physiotherapy modalities: supervised and unsupervised exercises, training, manual therapy, massage, hydrotherapy, electrotherapy, acupuncture, patient information, and educational programs
Intervention(s) characteristics	Mainly 30-60 min/day, 1-5 days/week
Professional that promoted the interventions	Not stated
Intervention(s) setting	Home, hospitals, Spas.
Control	Exercise programs, home exercise regimes, no intervention
Outcomes of interest (types and measuring instruments)	Pain (VAS pain), Stiffness (BASDAI), Spinal mobility (fingertip-to-floor distance - FFD), Physical function (modified Toronto Activities of Daily Living Questionnaire, BASFI), Patient global assessment, other relevant outcome measures
Methods of analysis	Meta-analysis + Narrative synthesis
Heterogeneity (I2)	NA
Effect size	Spinal mobility (Relative percentage differences -RPDs from 5-50%)<br>Pain (18%)<br>Physical function (24%)<br>PGA (27%)<br>Physical function (four points on a 33-point scale).
95% Confidence intervals	NA
P value	NA
Follow-up	2-32 weeks
Conclusions	Individual home-based or supervised exercise program is better than no intervention; Supervised group physiotherapy is better than home exercises; and that combined inpatient spa-exercise therapy followed by group physiotherapy is better than group physiotherapy alone.

DiRenzo et al., 2018	
Databases searched and language limits	Medline (PubMed), Embase, Web of Science, Cumulative Index of Nursing and Allied Health Literature (CINAHL, Ebsco), Cochrane Central Register of Controlled Trials, and PsycINFO (Ebsco).<br>English language only
Range of included studies	Through April 2018
Number, type of studies included and countries of origin	5 RCTs<br>USA, England, Norway, New Zealand.
Instruments used for bias appraisal	Cochrane Collaboration appraisal tools
Bias appraisal rating	Low bias
Participants characteristics (number, age, disease criteria, details)	Mainly RA patients, AS, PSA (n=399)<br>% Male 10.2-31.9%<br>Mean age 54<br>ACR criteria
Intervention(s)	Mindfulness-Based Interventions (Mindfulness-based stress Reduction, Mindful awareness and acceptance therapy, Vitality training program and Internal family systems)
Intervention(s) characteristics	Standardized program developed at the University of Massachusetts Medical School which consists of a 2.5 hour introductory session, 7 weekly 2.5 hour active session, and a 4-h silent retreat.<br>Sessions consist of a variety of mindfulness activities such as guided imagery, body scan, mindful eating, and gentle yoga.
Professional that promoted the interventions	Psychologists, health professionals and trained therapists
Intervention(s) setting	Not stated
Control	Education, CBT, usual care
Outcomes of interest (types and measuring instruments)	Depressive symptoms (SCL-90-R); Psychologic distress (SCL-90R:General Severity Index, GHQ-20); Anxiety (PANAS-X: Anxious Affect, State-Trait Anxiety Inventory); Self-efficacy and emotional processing (Arthritis Self-Efficacy Scale, Emotion Approach Coping Scale)
Methods of analysis	Meta-analysis + Narrative synthesis
Heterogeneity (I^2)	DAS28-CRP 0%<br>Pain 0%
Effect size	DAS28-CRP −0.44<br>Pain −0.58
95% Confidence intervals	DAS28-CRP −0.99 to 0.12<br>Pain −1.26 to 0.10
P value	DAS28-CRP 0.13<br>Pain 0.09
Follow-up	8-36 weeks
Conclusions	Mindfulness-Based Interventions may be a useful strategy to improve psychological distress in those with RA.

Dissanayake et al., 2010	
Databases searched and language limits	MEDLINE, EMBASE and the Cochrane Central Register of Controlled Trials<br>English language only
Range of included studies	1981 and 2009
Number, type of studies included and countries of origin	31 RCTs<br>Countries not stated
Instruments used for bias appraisal	Adapted Cochrane Collaboration appraisal tools
Bias appraisal rating	Score 4-9
Participants characteristics (number, age, disease criteria, details)	RA patients (n= 2021)<br>Not stated
Intervention(s)	Psychosocial interventions (Counselling, Psychotherapy, Mindfulness Meditation, CBT, Biofeedback, Relaxation therapy, Disclosure)
Intervention(s) characteristics	Duration – 1 days to 24 weeks
Professional that promoted the interventions	Not stated
Intervention(s) setting	Not stated
Control	Standard medical care, wait list.
Outcomes of interest (types and measuring instruments)	Pain, anxiety, depression, self-care, fatigue, physical function, psychological wellbeing, swelling, joint count, coping.
Methods of analysis	Narrative synthesis
Heterogeneity (I2)	NA
Effect size	Improved self-care, self-efficacy, knowledge, and greater use of health behaviours.<br>Improved self-efficacy in management of pain, function and general effects of RA.
95% Confidence intervals	NA
P value	NA
Follow-up	1-60 months
Conclusions	There is supportive evidence for the use of disclosure therapy, and CBT with maintenance therapy as adjunct therapies in patients with RA.

Du et al., 2011	
Databases searched and language limits	Medline and Embase<br>English language only
Range of included studies	1970 – March 2010
Number, type of studies included and countries of origin	19 RCTs<br>USA, UK, Netherlands, Switzerland, Canada, Hong Kong and Sweden
Instruments used for bias appraisal	Cochrane Collaboration appraisal tools
Bias appraisal rating	5 studies as A quality level, 14 studies as B quality level.
Participants characteristics (number, age, disease criteria, details)	Chronic musculoskeletal pain conditions include arthritis (OA, RA and PsA), and other (FM, back pain, shoulder pain, neck pain…) (n=6334)<br>Mean age 59
Intervention(s)	Self-management programs (including swimming sessions, relaxation, exercises, low impact land-based exercises, sessions on activities of daily living and education-discussion sessions, walking and Tai Chi)
Intervention(s) characteristics	Duration: 6 sessions-18 weeks
Professional that promoted the interventions	Pairs of lay leaders, physiotherapists, non-specific health care professionals
Intervention(s) setting	Home, remaining not stated
Control	Waiting-list control, education booklet, usual care, standard programs, no treatment
Outcomes of interest (types and measuring instruments)	Arthritis-related pain, Arthritis-related disability, Adverse event in self-management programs
Methods of analysis	Meta-analysis + Narrative synthesis
Heterogeneity (I2)	Pain at 4 months: 60%<br>Pain at 6 months: 0%<br>Pain at 12 months: 0%<br>Disability at 4 months: 39%
Effect size	Pain at 4 months: -0.23<br>Pain at 6 months: -0.29<br>Pain at 12 months: -0.14<br>Disability at 4 months: -0.06
95% Confidence intervals	Pain at 4 months: -0.36 to -0.10<br>Pain at 6 months: -0.41 to 0.17<br>Pain at 12 months: -0.23 to -0.04<br>Disability at 4 months: -0.17 to 0.05
P value	Pain at 4 months: <0.005<br>Pain at 6 months:<0.005<br>Pain at 12 months: 0.008<br>Disability at 4 months: 0.26
Follow-up	2-36 months
Conclusions	Self-management is a safe, community-based and effective way for patients with arthritis to manage pain and disability.<br>Core skills of self-management should be delivered using multiple approaches.

Knittle et al., 2010		
Databases searched and language limits	PsycINFO, MEDLINE, the central catalog of Dutch libraries<br>English or Dutch	
Range of included studies	1981-2007	
Number, type of studies included and countries of origin	27 RCTs<br>US, UK, Netherlands, Canada, Sweden, Austria	
Instruments used for bias appraisal	Adapted 29-item version of the Cochrane Collaboration Depression Anxiety, and Neurosis Review Group scale	
Bias appraisal rating	Scores between 21-42	
Participants characteristics (number, age, disease criteria, details)	Number of participants and characteristics not stated<br>RA patients (ACR criteria or defined by the ARA)	
Intervention(s)	Relaxation, Cognitive pain management strategies, Self-Management Program, Cognitive–behavioural therapy, Education, Contracting, Goal setting, Provision of feedback, Cognitive restructuring, Joint protection, Problem solving, Exercise, Coping, Group counselling, Guided imagery, Self-instruction, Range of motion exercises, Mindfulness	
Intervention(s) characteristics	Frequency 1-5 times per week<br>Duration 30 min-2.5 hours<br>Intensity not stated	
Professional that promoted the interventions	PhD student, Layperson, Psychologist, Medical doctor, Occupational therapist, Physical therapist, Nurse, Counsellor, Dietician	
Intervention(s) setting	Home, majority unstated	
Control	Education or no intervention	
Outcomes of interest (types and measuring instruments)	Pain (VAS)<br>Disability (HAQ)<br>Depressive and anxiety symptoms (HADS, STAI, BDI, ZDS, DS, CES-D, SCL90-R)	
Methods of analysis	Meta-analysis + Narrative synthesis	
Heterogeneity (I^2)	Pain 0%, Disability 60.26%, Depressive symptoms 46.95%, Anxiety 0%	
Effect size	Pain g= 0.18<br>Disability g= 0.32,<br>Physical activity g= 0.45<br>Depressive symptoms g=0.23<br>Anxiety g=0.17.	At follow-up:<br>Pain g=0.13<br>Disability g= 0.15<br>Physical activity g=0.36<br>Depressive symptoms g= 0.32<br>Anxiety g=0.12.
95% Confidence intervals	Pain 0.08 to 0.29<br>Disability 0.13 to 0.51<br>Physical activity 0.11 to 0.82<br>Depressive symptoms 0.05 to 0.50<br>Anxiety -0.06 to 0.30	At follow-up<br>Pain 0.01 to 0.26<br>Disability 0.002 to 0.28<br>Physical activity 0.05 to 0.66<br>Depressive symptoms 0.16 to 0.48<br>Anxiety -0.06 to 0.30.
P value	Pain 0.006<br>Disability 0.001<br>Physical activity 0.01<br>Depressive symptoms 0.01<br>Anxiety 0.20.	At follow-up Pain 0.006<br>Disability 0.05<br>Physical activity 0.02<br>Depressive symptoms <0.005<br>Anxiety 0.02.
Follow-up	2-16 weeks	
Conclusions	Psychological interventions are beneficial for many patients with RA, particularly when it comes to increasing physical activity levels.	

Lopes et al., 2016	
Databases searched and language limits	PubMed/MEDLINE<br>English language only
Range of included studies	January 2004 - January 2014
Number, type of studies included and countries of origin	8 RCTs<br>Not stated
Instruments used for bias appraisal	PEDro
Bias appraisal rating	Score 5-7
Participants characteristics (number, age, disease criteria, details)	AS patients (n=345)
Intervention(s)	Exercise programs
Intervention(s) characteristics	20 min-1h per day, 8-16 weeks
Professional that promoted the interventions	Not stated
Intervention(s) setting	Home, remaining not stated
Control	No treatment, education session, conventional exercise
Outcomes of interest (types and measuring instruments)	BASMI, BASFI, BASDAI, FFD, VAS, BDI, BAS-G, ASQOL
Methods of analysis	Narrative synthesis
Heterogeneity (I2)	NA
Effect size	NA
95% Confidence intervals	NA
P value	NA
Follow-up	12 weeks-12 months
Conclusions	Exercise group programmes are more effective than home-based ones in patients with AS.<br>It could be advantageous to carry out home-based exercise programs than the absence of any exercise program.

Mudano et al., 2019	
Databases searched and language limits	CENTRAL, MEDLINE, Embase, and clinical trial registries<br>Not stated
Range of included studies	2002 to September 2018.
Number, type of studies included and countries of origin	7 RCTs (n=345)<br>China, South Korea, and the USA
Instruments used for bias appraisal	Cochrane Collaboration appraisal tools
Bias appraisal rating	The quality of the evidence was low or very low for all major outcomes.
Participants characteristics (number, age, disease criteria, details)	RA adult patients<br>Mostly women<br>Age ranging from 16 to 80 years<br>ACR criteria
Intervention(s)	Exercise programs with Tai Chi instruction, or exercises that incorporated principles of Tai Chi training
Intervention(s) characteristics	Duration: 8 to 12 weeks
Professional that promoted the interventions	Not stated
Intervention(s) setting	Hospital
Control	Non-exercise or alternative exercise method
Outcomes of interest (types and measuring instruments)	Pain, disease activity (DAS-28-ESR), function (HAQ), and radiographic progression
Methods of analysis	Meta-analysis + Narrative synthesis
Heterogeneity (I^2)	Pain 0%<br>DAS-28-ESR NA<br>HAQ 82%
Effect size	Pain: MD -2.15<br>DAS-28-ESR: MD -0.4<br>HAQ: MD -0.33<br>Non significance for number of tender and swollen joints, grip strength.
95% Confidence intervals	Pain -3.19 to -1.11<br>DAS-28-ESR -1.1 to 0.3<br>HAQ -0.79 to 0.12
P value	Pain <0.005<br>DAS-28-ESR 0.26<br>HAQ 0.15
Follow-up	8-24 weeks
Conclusions	It is uncertain whether Tai Chi has any effect on clinical outcomes (joint pain, activity limitation, function) in RA, and important effects cannot be confirmed or excluded, since all outcomes had very low-quality evidence.

Pécourneau et al. 2018	
Databases searched and language limits	Medline via PubMed and Cochrane Library<br>English or French language only
Range of included studies	January 1980 to May 2017
Number, type of studies included and countries of origin	8 RCTs (n=331)<br>Spain, Turkey, Italy, Norway
Instruments used for bias appraisal	Jadad score
Bias appraisal rating	Mean Jadad score 2.1 (range 1 to 3)
Participants characteristics (number, age, disease criteria, details)	AS adult patients (as by ASAS criteria for axial spondyloarthritis or the modified New York criteria)
Intervention(s)	Modalities of exercise: specific exercise (swimming, aerobic etc.), home-based exercise program, and supervised exercise by healthcare professionals.
Intervention(s) characteristics	Frequency: 1-6 per week<br>Session duration: 20-90 min<br>Program duration: 3-16 weeks
Professional that promoted the interventions	Physiotherapist, certified coach.
Intervention(s) setting	Home, pool, sports room
Control	Physical therapy, usual care, education
Outcomes of interest (types and measuring instruments)	BASDAI, BASFI
Methods of analysis	Meta-analysis + Narrative synthesis
Heterogeneity (I^2)	BASDAI: 69%<br>BASFI: 0%
Effect size	BASDAI: WMD -0.90<br>BASFI: WMD -0.72
95% Confidence intervals	BASDAI: -1.52 to -0.27<br>BASFI: -1.03 to -0.40
P value	BASDAI: 0.005<br>BASFI: <0.005
Follow-up	Not stated
Conclusions	Exercise programmes improve disease activity and body function in AS.

Marques A, *et al. RMD Open* 2021; 7:e001647. doi: 10.1136/rmdopen-2021-001647

Riemsma et al., 2003	
Databases searched and language limits	MEDLINE, EMBASE and PsycINFO and the Cochrane Controlled Trials Register<br>No language restriction
Range of included studies	1966 forward to September 2002
Number, type of studies included and countries of origin	50 RCTs<br>Countries not stated
Instruments used for bias appraisal	Adapted Jadad score
Bias appraisal rating	Range 0 to 7
Participants characteristics (number, age, disease criteria, details)	Adult RA patients<br>Mean age of 56 years<br>53.5% female<br>ACR criteria
Intervention(s)	Patient Education, Information Only, Counselling, Behavioural Treatment
Intervention(s) characteristics	Developed over several sessions and periods of different duration
Professional that promoted the interventions	Not stated
Intervention(s) setting	Not stated
Control	Symptom monitoring, no-intervention, waiting list controls, standard rheumatologic care, lecture (90 min) on pain management
Outcomes of interest (types and measuring instruments)	Pain (VAS), psychological status, anxiety, and depression (HADS, CES-D, ZSRDS), AIMS, Disability (HAQ), Disease activity (ESR, CRP)
Methods of analysis	Meta-analysis + Narrative synthesis
Heterogeneity (I^2)	Disability 20%<br>Joint counts 0%<br>PGA 0%<br>Psychological status 0%<br>Depression 0%
Effect size	Disability: SMD = -0.17<br>Joint counts: SMD = -0.13<br>PGA: SMD = -0.28<br>Psychological status: SMD= -0.15<br>Depression: SMD = -0.14<br>No statistical significance was found for pain, anxiety, disease activity and for final follow-up in patient education.<br>No statistical significance for Information Only, Counselling, Behavioural Treatment at the beginning and end of the follow-up.
95% Confidence intervals	Disability -0.25 to -0.09<br>Joint counts -0.24 to -0.01<br>PGA -0.49 to -0.07<br>Psychological status -0.27 to -0.04<br>Depression -0.23 to -0.05
P value	Disability <0.005<br>Joint counts 0.03<br>PGA 0.008<br>Psychological status 0.01<br>Depression 0.004
Follow-up	3-14 months
Conclusions	Patient education as provided in the studies reviewed here had small short-term effects on disability, joint counts, patient global assessment, psychological status and depression. There was no evidence of long-term benefits in adults with RA.

Randomised controlled trials

Barlow et al., 1998 & Barlow et al., 1997	
Participants characteristics (number, age, disease criteria, details)	RA patients (n=108)<br>80% woman<br>≈ 60 years<br>≈ Disease duration 12 years
Intervention(s)	Patient education RA leaflets
Intervention(s) characteristics	The 'Rheumatoid Arthritis' leaflet used was one of a range of leaflets produced by the Arthritis & Rheumatism Council.
Professional that promoted the interventions	Doctors or nurse practitioners
Intervention(s) setting	Hospital
Control	Patients read a leaflet (other than the ARC leaflet)
Outcomes of interest (types and measuring instruments)	Knowledge Scale based on the content of the ARC 'Rheumatoid Arthritis' leaflet, modified Health Assessment Questionnaire (HAQ), pain and fatigue visual analogue scales (VAS), the Hospital Anxiety and Depression Scale (HADS), the Arthritis Self-Efficacy: Pain (ASE: Pain) and the Arthritis Self-Efficacy: Other Symptoms (ASE: Other Symptoms) scales.
Methods of analysis	Correlational analysis
Effect size	Not stated
95% Confidence inter.	Not stated
P value	Increase in knowledge ($p < 0.005$)<br>Decrease in pain ($p=0.01$) and in depression ($p=0.05$).<br><br>At follow-up there are no differences.
Follow-up	3 weeks to 6 months
Conclusions	Patient education leaflets can be an effective means of increasing knowledge amongst patients with RA and appear to generate a sense of reassurance. Moreover, change in knowledge can be maintained over periods of months rather than weeks.

Barsky et al., 2010	
Participants characteristics (number, age, disease criteria, details)	RA patients (n=168)<br>≈ 86% female<br>≈ 53 years<br>≈ Disease duration 13 years<br>ACR criteria
Intervention(s)	Cognitive-behaviour therapy (CBT), relaxation response training (RR), or arthritis education
Intervention(s) characteristics	The CBT consisted of 12 sessions of 60 to 75-minutes. It was a slightly modified and shortened version of the treatment program of Bradley and co-workers. Techniques taught included problem-solving and goal setting, relabelling and reframing of symptoms, enhanced awareness of pain behaviours, activity pacing, and distraction and attention refocusing. Homework was given at the end of each session and reviewed at the beginning of the following session.
Professional that promoted the interventions	Not stated
Intervention(s) setting	Hospital
Control	Arthritis education
Outcomes of interest (types and measuring instruments)	Rheumatoid Arthritis Symptom Questionnaire (RASQ), Grip strength, Walking time, Rand Mental Health Inventory, Arthritis Impact Measurement Scale (AIMS-2)
Methods of analysis	Correlational analysis
Effect size	Effect sizes ranged between 0.26 and 0.35.
95% Confidence inter.	Not stated
P value	Pain improved significantly at 12 months in the RR and AE groups and showed a nonsignificant positive trend with CBT. Other RA symptoms improved significantly with CBT and AE and showed a nonsignificant trend with RR.<br>There were no significant differences in the outcomes across the 3 treatment groups. When the results for all 3 groups were aggregated, significant benefits were found for pain, other RA symptoms, self-care activities, and social activities.
Follow-up	6 and 12 months
Conclusions	These 3 psychosocial treatments were beneficial, with treatment effect sizes in the small to moderate range. The effects appeared immediately after treatment and were generally sustained at long-term follow-up. These benefits were achieved over and above those resulting from medical management. These treatments constitute an effective augmentation to standard medical therapy for RA patients.

Basler et al., 1991		
Participants characteristics (number, age, disease criteria, details)	Patients with ankylosing spondylitis (n=39)<br>56% men<br>≈ 45 years	
Intervention(s)	Cognitive-behavioural therapy	
Intervention(s) characteristics	Training in progressive muscle relaxation, cognitive restructuring, attention related techniques, and pleasant activity scheduling.	
Professional that promoted the interventions	Not stated	
Intervention(s) setting	Self-help organization	
Control	Waiting-list controls	
Outcomes of interest (types and measuring instruments)	Pain<br>Impairment<br>Daily mood<br>Depression<br>Anxiety<br>Psychophysiological complains<br>Sleep disturbances	
Methods of analysis	Correlational analysis	
Effect size	At post-treatment<br>Pain 0.06<br>Impairment 0.07<br>Daily mood 0.07<br>Depression 0.43<br>Anxiety 0.35<br>Psychophysiological complains 0.75<br>Sleep disturbances 0.41	At follow-up<br>Pain 0.07<br>Impairment 0.13<br>Daily mood 0.01<br>Depression 0.44<br>Anxiety 0.60<br>Psychophysiological complains 0.76<br>Sleep disturbances 0.62
95% Confidence inter.	Not stated	
P value	Not stated	
Follow-up	6 months	
Conclusions	Reductions of pain intensity, anxiety and psychophysiological symptoms were maintained at 12-month follow-up. Although pain reduction was statistically significant, it did not exceed 14% in pain dairy. The more important aspect of the treatment appears to be emotional stabilization and increased feelings of well-being.	

El Miedany et al., 2012	
Participants characteristics (number, age, disease criteria, details)	RA patients (n=147)<br>≈ 71.6% female<br>≈ 53 years<br>≈ Disease duration 11 years<br>ACR criteria
Intervention(s)	Arthritis education and self-management
Intervention(s) characteristics	Patients were encouraged to discuss their problems and set their own goals.<br>They were also encouraged to view the scores recorded of their former self-reported outcome measures as well as to discuss the changes in their disease activity parameters, co-morbidity risks, functional disability, and quality of life.<br>The patients were taught what to anticipate or what was likely to happen and guided with the skills needed for self-care and decision making regarding the next step in the joint fitness programme.
Professional that promoted the interventions	Rheumatologist
Intervention(s) setting	Not stated
Control	Standard management
Outcomes of interest (types and measuring instruments)	Patients' adherence to their medications, disease activity score (DAS-28) and PROMs domains: pain score, patient global assessment, functional disability, quality of life and self-helplessness.
Methods of analysis	Correlational analysis
Effect size	At the 18-month follow-up, both the self-management and cognitive behavioural therapy intervention demonstrated improvement for disease activity (effect size 1.4 and 1.2, respectively).
95% Confidence inter.	Not stated
P value	The integration of patient education and PROMs led to a significant greater reduction of disease activity parameters, DAS-28 score, as well as improvement of the patients' adherence to therapy (p<0.01).<br>The improvement of disease activity parameters were associated with the improvement in functional disability and quality of life scores.
Follow-up	18 months
Conclusions	The integration of patient education and PROMs succeeded in improving self-perceived health as well as disease activity.<br>The patient education for patients with inflammatory arthritis is feasible in the standard clinical practice.

Evers et al., 2002	
Participants characteristics (number, age, disease criteria, details)	Early RA patients (n=59)<br>≈ 71 % Female<br>≈ 53.5 years<br>≈ Disease duration 3.1 years<br>ACR Criteria
Intervention(s)	Cognitive-behavioural treatment
Intervention(s) characteristics	The treatment modules were developed from standardized CBT protocols.<br>The pain and function disability module consisted of progressive relaxation, attention diversion, stimulation of physical exercising in daily life in the face of the current physical condition, activity pacing, problem-solving, adjustment of goal-setting to the current physical condition, identification of pain-provoking cues in daily life, and cognitive restructuring of dysfunctional pain cognitions.<br>The fatigue module included activity-pacing, adjustment of goal setting to the current physical condition, setting priorities and structured planning of daily activities and time off, and cognitive restructuring of activity demands.<br>The negative mood module consisted of problem-solving, cognitive restructuring of depressogenic and anxious cognitions, identification of stress-provoking cues in daily life, stimulating pleasurable activities and restructuring of goal-setting in the face of the current physical condition, emotional processing of the changes RA has brought about in daily life and finding benefits.<br>The social module finally included identification of social stress provoking cues in daily life, cognitive restructuring of social anxious cognitions, stimulating social activities in the face of the current physical condition, and social skills training including help-seeking behaviour and communication about RA.<br>In all treatment modules, the final booster session dealt with relapse prevention and further improvement of the attained goals.
Professional that promoted the interventions	Two therapists trained in the treatment modules and supervised by a cognitive-behaviour supervisor.
Intervention(s) setting	Not stated
Control	Standard care
Outcomes of interest (types and measuring instruments)	Disease activity - DAS28_ESR<br>Functional disability - Mobility and Self-care scales of the Impact of Rheumatic Diseases on General Health and Lifestyle (IRGL)<br>Pain - IRGL Pain scale<br>Fatigue - Fatigue scale (eight items)<br>of the Checklist Individual Strength<br>Psychological functioning - IRGL<br>Anxiety and Negative Mood scales
Methods of analysis	Chi-square analyses for categorical variables and Student's t-test for continuous variables<br>Multivariate analyses
Effect size	Effect sizes were 0.55 and 0.48 for fatigue and 0.51 and 0.55 for depression at post-treatment and follow-up assessment, respectively.<br>In addition, small to medium effects were found for negative mood, anxiety and perceived support at post-treatment and follow-up assessment (0.44 and 0.43 for negative mood, 0.57 and 0.28 for anxiety, and 0.16 and 0.38 for perceived support at post-treatment and follow-up assessment, respectively
95% Confidence Inter.	Not stated
P value	Beneficial effects of CBT on physical, psychological and social functioning. Specifically, fatigue and depression were significantly reduced at post-treatment and at the 6-month follow-up in the CBT condition in comparison to the control condition, while perceived support increased at follow-up assessment. In addition, helplessness decreased at post-treatment and follow-up assessment, active coping with stress increased at post-treatment, and compliance with medication increased at follow-up assessment in the CBT condition in comparison to the control condition.
Follow-up	6 months
Conclusions	Results indicate the effectiveness of tailor-made CBT for patients at risk in relatively early RA, and supply preliminary support for the idea that customizing treatments to patient characteristics may be a way to optimize CBT effectiveness in RA patients

Freeman et al., 2002	
Participants characteristics (number, age, disease criteria, details)	Early RA patients (n=54)<br>≈ female not stated<br>≈ age not stated<br>≈ Disease duration 4.5 months<br>ACR Criteria
Intervention(s)	A program with cognitive-behavioural arthritis education
Intervention(s) characteristics	The cognitive-behavioural arthritis education programme used the health belief model and theory of self-efficacy as a theoretical framework.<br>Participants were given accurate information regarding disease pathology and treatment with emphasis on prevention of joint pain,<br>joint deformity and loss of joint function followed by reassurance that treatment could be effective
Professional that promoted the interventions	Not stated
Intervention(s) setting	Clinics
Control	Standard arthritis education programme
Outcomes of interest (types and measuring instruments)	Physical Function Subscale of the Arthritis Impact Measurement Scale Two (AIMS2)<br>Secondary outcome measures included: ESR, duration of early morning stiffness (minutes) and DAS28
Methods of analysis	Wilcoxon signed ranks and Friedman<br>ANOVA, Mann–Whitney (U) and chi square tests
Effect size	Not stated
95% Confidence interval	Not stated
P value	Baseline analysis revealed that the control group had significantly better levels of functional ability (U = 185; p = 0.009) and lower levels of helplessness (U = 168; p = 0.002) prior to intervention.<br>This difference remained unchanged three months later.<br>Six months following the intervention no significant differences were noted between the groups for any measure of health status.<br>There were no significant changes in health status over time in either group.
Follow-up	6 months
Conclusions	Attending a cognitive-behavioural arthritis education programme had no significant effect on the health status of individuals newly diagnosed with RA. The move to early use of these programmes should be examined further, with a larger sample size and longer duration of follow-up

Giraudet-Le Quintrec et al., 2007	
Participants characteristics (number, age, disease criteria, details)	RA patients (n=208)<br>≈ 97% female<br>≈ 55 years<br>≈ Disease duration 13 years<br>ACR Criteria
Intervention(s)	The programme employed self-efficacy principles to reduce pain and stress at home, and behavioural modification techniques to change behaviours and improve quality of life by modifying psychological and social contacts
Intervention(s) characteristics	The interactive multidisciplinary education program consisted of passive information on the disease, on medical treatment, and on lifestyle advice concerning diet, but also included information on active coping strategies, relaxation, and physical exercise, with the teaching of an exercise program to be followed at home. Sessions were conducted on Thursdays for 6 hours for 8 consecutive weeks.<br>After 6 months, patients attended a 4-hour "booster" session.
Professional that promoted the interventions	Rheumatologist, rehabilitation specialist, dietician, social assistant, 2 nurses, 2 physiotherapists and 2 occupational therapists
Intervention(s) setting	Hospital
Control	Usual medical care.
Outcomes of interest (types and measuring instruments)	Functional score – Health Assessment Questionnaire (HAQ)<br>Disease Activity Score (DAS28)<br>Hospital Anxiety and Depression Scale (HADS)<br>Arthritis Helplessness Index (AHI) score for Coping<br>Quality of life using the EMIR<br>Arthritis Impact Measurement Scale (AIMS2)<br>Functional Assessment of Chronic Illness Therapy – Fatigue scale (FACIT-F)<br>Drug compliance<br>Satisfaction with the program<br>Quality of information for each aspect of the program provided by leaflets and/or educational classes
Methods of analysis	Student's t-test
Effect size	Not stated
95% Confidence inter.	Not stated
P value	After 1 year, no statistically significant difference was observed between the 2 groups in change in HAQ score: –0.04 ± 0.46 (education group) vs –0.06 ± 0.47 (control group) (p = 0.79).<br>Statistically significant differences were found in 3 domains, all of which were better for the group attending the education sessions:<br>Patient coping (–1.22 ± 5.55 vs –0.22 ± 3.81; p = 0.03),<br>Knowledge (3.42 ± 4.73 vs 0.73 ± 3.78; p < 0.0001)<br>Satisfaction (10.07 ± 11.70 vs 5.72 ± 13.77; p = 0.02)
Follow-up	12-month
Conclusions	Despite improvements in patient coping, knowledge, and satisfaction, the education program was not found to be effective at 1 year.<br>There may have been methodological problems relating to the sensitivity of questionnaires and patient selection, and tailored educational interventions should be considered.

Hammond et al., 2008	
Participants characteristics (number, age, disease criteria, details)	Participants with RA or early IA or PsA (n=167)<br>≈ 64.7% female<br>≈ 55.4 years<br>≈ Disease duration 7.3 years
Intervention(s)	Group modular cognitive–behavioural approach programme (the LMAP)
Intervention(s) characteristics	This included 2 modules, each with four 2.5h, and one 2-h review meeting. Module manuals were developed and Arthritis Research Campaign and Arthritis Care booklets were provided. Each meeting included self-monitoring, skills training to follow individually determined home activity and exercise programmes.<br>Meeting 1 discussed RA and PsA, health beliefs, personal impact of arthritis, understanding multiple factors affecting symptoms, attitudes, personal experiences of what helps, self-management methods and motivating for change.<br>Meetings 2–4 focused on applying ergonomic approaches to reduce pain, hand exercises, fatigue management and benefits of splints.<br>In Module 2, discussions focused on participants' exercise beliefs, barriers and problem-solving these.<br>Each module was delivered to facilitate continuing discussion of themes, negotiating home programmes and weekly review of progress with goals.
Professional that promoted the interventions	One rheumatology OT, one community OT and one rheumatology PT
Intervention(s) setting	Not stated
Control	Group information-focused (or standard) programme
Outcomes of interest (types and measuring instruments)	Physical status – HAQ<br>Psychological status: the RA Self-efficacy (RASE) Scale; Arthritis Self-efficacy Scale (ASES)<br>Self-management - Arthritis Stages of Change Questionnaire.<br>Health care use - self-reported number of visits to physicians during the last 6 months; current medication
Methods of analysis	x^2 or unpaired t-tests as appropriate
Effect size	Not stated
95% Confidence inter.	Not stated
P value	At 6 months, the behavioural group had better pain (P.0.01), fatigue (P.0.01), functional ability (P.0.05) and self-efficacy (P.0.01) scores and greater use of health behaviours.<br>At 12 months, they continued to have better pain (P.0.03), self-efficacy (P.0.001) and psychological status (P.0.0001) scores and greater use of some health behaviours
Follow-up	12 months
Conclusions	Attending a modular behavioural education programme is effective for at least 1 year in enabling people with RA and PsA to reduce pain, improve psychological status and self-manage their condition.

Hewlett et al., 2011	
Participants characteristics (number, age, disease criteria, details)	RA patients (n=126)<br>≈ 73.2% female<br>≈ 59.2 years<br>≈ Disease duration 14 years<br>ACR Criteria
Intervention(s)	Group cognitive behavioural therapy (CBT) for fatigue self-management
Intervention(s) characteristics	6 *2 h sessions (weeks 1–6), with a 1 h consolidation session (week 14).<br>Topics likely to improve fatigue self-management were included. Thoughts, feelings and behaviours related to fatigue were addressed using Socratic (reflective) questioning and guided discovery to enable patients to work out links themselves.<br>Problem-solving, goal setting, self-monitoring of activity/rest and energy management, aimed to help patients turn cognitive and behavioural changes into improved well-being. Goal setting occupied the second hour of sessions, each clinician taking half the group to help patients set and review personal cognitive or behavioural goals. Programme homogeneity across groups was maintained through standardised topics, tools, metaphors and handouts, delivered by the same clinicians.
Professional that promoted the interventions	Clinical psychologist and a specialist occupational therapist
Intervention(s) setting	Hospital
Control	Receiving fatigue information alone
Outcomes of interest (types and measuring instruments)	Fatigue - (Multi-dimensional<br>Assessment of Fatigue (MAF); VAS 0–10)<br>Disability - Health Assessment Questionnaire (HAQ, 0–3)<br>Personal Impact - HAQ (0–9)<br>Wider outcomes - RA Quality-of-Life scale (0–30), Hospital Anxiety and Depression Scale (0–21), Arthritis Helplessness Index (5–30), RA Self-Efficacy scale (RASE, 28–140) and a sleep quality question (very good, fairly good, fairly bad, very bad)
Methods of analysis	Multivariable linear regression models
Effect size	The standardised effect size for MAF was 0.59 (95% CI 0.15 to 1.03) and 0.77 for fatigue impact VAS (95% CI 0.33 to 1.21) in favour of the CBT intervention
95% Confidence inter.	Above
P value	At 18 weeks CBT participants reported better scores than control participants for fatigue impact: MAF 28.99 versus 23.99 (adjusted difference −5.48, 95% CI −9.50 to −1.46, p=0.008); VAS 5.99 versus 4.26 (adjusted difference −1.95, 95% CI −2.99 to −0.90, p<0.001).
Follow-up	4.5 months
Conclusions	Group CBT for fatigue self-management in RA improves fatigue impact, coping and perceived severity, and well-being.

Hill et al., 2001	
Participants characteristics (number, age, disease criteria, details)	RA patients (n=100)<br>≈ 74% female<br>≈ 62.5 years<br>≈ Disease duration 12.5 years<br>ACR Criteria
Intervention(s)	Patient education programme (PE) would improve rates of adherence to a slow acting antirheumatic drug and to assess any subsequent effect on patient outcome
Intervention(s) characteristics	30-minute appointment at monthly intervals over a six-month period comprising seven visits. Patients in the intervention cohort received their PE.<br>The programme comprised information about the types of drugs used for RA, the disease process, physical exercise, joint protection, pain control, and coping strategies. Written information, including a DPA drug information leaflet developed specially for the study, was provided as back up.
Professional that promoted the interventions	Rheumatology nurse practitioner
Intervention(s) setting	University teaching hospital
Control	Standard management
Outcomes of interest (types and measuring instruments)	Adherence - pharmacological marker<br>Clinical assessments—articular index (AI), morning stiffness, and pain score
Methods of analysis	Logistic regression analysis
Effect size	Not stated
95% Confidence intervals	Not stated
P value	Education group demonstrate to be significantly more adherent on more occasions than the control group (p<0.05). Patterns of adherence over time showed that at 12 weeks 86% (38/ 44) of those in the EG compared with 64% (29/45) of the CG remained adherent (p=0.01). These trends continued and by the end of the study 85% (29/34) of the EG compared with 55% (23/42) of the CG were taking their DPA as prescribed.
Follow-up	6 months
Conclusions	Patient Education significantly increased adherence to DPA and its effects persisted over a period of six months. No additional clinical benefit was detected in the EG in comparison with the CG.

Knittle et al., 2013	
Participants characteristics (number, age, disease criteria, details)	RA patients (n=78)<br>≈ 67% female<br>≈ 63 years<br>≈ Disease duration not stated<br>ACR Criteria
Intervention(s)	Education session plus a motivational interview from a physical therapist and two self-regulation coaching sessions from a rheumatology nurse.
Intervention(s) characteristics	In week 1, both control and treatment participants attended a group educational session (3-7 people).<br>In week 2, treated patients received a one-to-one education session of 45 min. Patients weighed the pros and cons of (re-)engaging in PA, and links were made for physically active lifestyle and settling of long-term goals.<br>In weeks 4 and 5, one to one coaching sessions (40-60min) to patients in the treatment group were made to enhance fidelity of intervention delivery, followed the structure of a workbook developed for this study.<br>In weeks 6, 12 and 18, patients in the treatment group received a follow-up phone call to discuss the patient's efforts in self-regulating physical activity.
Professional that promoted the interventions	Rheumatology nurse<br>Physical therapist
Intervention(s) setting	Hospital
Control	Control group—which received a group-based patient education session led by a physical therapist
Outcomes of interest (types and measuring instruments)	Leisure-time physical activity - Short Questionnaire to Assess Health-Enhancing Physical Activity<br>Self-efficacy for PA - 18-item questionnaire from Bandura<br>Autonomous motivation for PA – three items from the Treatment Self-Regulation Questionnaire<br>Disease activity - Rheumatoid Arthritis Disease Activity Index (RADAI)<br>Functional status - 20-item disability scale of the Health Assessment Questionnaire (HAQ)<br>Depressive symptoms - 6 items from the Brief Symptom Inventory (BSI)<br>Fatigue - 20-item Checklist of Individual Strengths (CIS-20)
Methods of analysis	Mixed model repeated measures analyses
Effect size	Leisure time PA:<br>Post treatment p =0.29<br>6 months p =0.29
95% Confidence intervals	Leisure time PA:<br>64 (−12.2, 140.2) Post treatment<br>84 (−2.9, 170.9) 6 months
P value	Over the 32 weeks of the study, there were significant main effects (group * time) on leisure time PA (F =4.01; p =0.022), days per week with 30 min of PA (F =4.39; p =0.016), total self-efficacy (F =5.18; p =0.001) and autonomous motivation (F =7.16; p =0.008); but not disease activity (F =2.17; p =0.121), functional status (F =0.64; p =0.530), depressive symptoms (F =1.35; p =0.266) or fatigue (F =0.43; p = 0.651).<br>No significant effects were found for disease activity, functional status, depressive symptoms or fatigue.
Follow-up	6 months
Conclusions	Combining motivation- and action-focused intervention approaches improved PA-related cognitions and led to improved uptake and maintenance of leisure-time PA

Lumley et al., 2014	
Participants characteristics (number, age, disease criteria, details)	Early RA patients (n=264)<br>≈ 81% female<br>≈ 55 years<br>≈ Disease duration not stated<br>ACR Criteria
Intervention(s)	3 intervention groups<br>• Written emotional disclosure (WED) + coping skills training<br>• WED + control training<br>• Coping skills training (CST) + control writing
Intervention(s) characteristics	Patients started their assigned writing condition, which continued for the next week, after which they immediately began their assigned training condition, with eight sessions held at weekly intervals.<br>WED - Session 1, patients were instructed to identify a stressful and to write about their; Session 2, patients were asked to continue writing stressful experience; Session 3, patients were instructed to try to find meaning from their stressful experience and to write about anything they learned from their experience; Session 4, patients were asked to write about how they coped with their stressful experience.<br>WED control - Session 1, patients wrote about how they spent and managed their time during the prior week; Session 2, patients wrote about their eating behaviours during the current day and reviewed their eating over recent days; Session 3, patients wrote about physical activity behaviours during the past week; Session 4, patients detailed their sleep over the past week.<br>CST- Session 1, patients learned progressive muscle relaxation; Session 2, relaxation was reviewed and pleasant activity scheduling was taught; Session 3, assertive communication skills were introduced and the use of applied relaxation in daily life was taught; Session 4, communication skills training was continued and cognitive restructuring introduced; Session 5, cognitive restructuring, was continued and activity-rest cycling was also covered; Session 6, activity-rest cycling was reviewed and distraction skills were taught; Session 7, patients reviewed all of the skills and learned problem solving; Session 8, relapse prevention was addressed.<br>CST Control<br>Session 1 characteristics of RA; Session 2, joint anatomy and physiology, and signs and symptoms of RA; Session 3, diagnosis and prognosis of RA, goals of treatment,<br>overview of all treatments; Session 4, the immune system and RA-specific medications; Session 5, pain assessment, analgesics, and other pain medications; Session 6, complementary and alternative medicine interventions, dietary supplements, nutrition, weight control, exercise; Session 7, surgery, physical modalities, physical and occupational therapy, adaptive devices; and Session 8, shared management of RA, clinical trials for RA, and resources for patients, including Internet skills.
Professional that promoted the interventions	CST - advanced doctoral students or postdoctoral fellows in clinical psychology<br>CTS control - primarily nurses.
Intervention(s) setting	Hospital
Control	Control writing + Control training
Outcomes of interest (types and measuring instruments)	Disease activity – 32 joints + PGA<br>Pain - self-report Arthritis Impact Measurement Scales-2 (AIMS2)<br>Physical disability - AIMS2 subscales<br>Psychological symptoms - AIMS2 subscales<br>Walking speed - 50-foot hallway<br>Inflammatory activity - CRP
Methods of analysis	ANCOVA
Effect size	The benefits of CST are the clearest.<br>The between-group effect sizes of this intervention on these outcomes ranged from 0.23 to 0.37 standard deviations
95% Confidence int.	Not stated
P value	Hierarchical linear modelling of treatment effects over the follow-up period, and analyses of covariance at each assessment point, revealed no interactions between writing and training; however, both interventions had main effects on outcomes, with small effect sizes. Compared with control training, CST decreased pain and psychological symptoms through 12 months. The effects of WED were mixed: Compared with control writing, WED reduced disease activity and physical disability at 1 month only, but WED had more pain than control writing on 1 of 2 measures at 4 and 12 months
Follow-up	12 months
Conclusions	The combination of WED and CST does not improve outcomes, perhaps because each intervention has unique effects at different time points. CST improves health status in RA and is recommended for patients, whereas WED has limited benefits and needs strengthening or better targeting to appropriate patients

Manning et al., 2014	
Participants characteristics (number, age, disease criteria, details)	Early RA patients (n=108)<br>≈ 76% female<br>≈ 55 years<br>≈ Disease duration 1.2 years<br>ACR Criteria
Intervention(s)	A brief, supervised education, self-management, and global upper extremity exercise training (EXTRA) program, supplementing a functional home exercise regimen, aimed at improving global upper extremity disability in people with RA
Intervention(s) characteristics	EXTRA program was refined to comprise 4 supervised group (4–6 participants per group) education, self-management, and global upper extremity exercise training sessions (delivered twice weekly for the first 2 weeks of the intervention) supplementing a functional daily home exercise regimen.<br>The supervised sessions commenced with a 15-minute interactive discussion/seminar designed to increase participants' knowledge of RA and exercise, self-efficacy, and disease self-management, and facilitate uptake and longer-term exercise participation. Behaviour change strategies were integrated into the seminars and reviewed, where necessary, in subsequent sessions.
Professional that promoted the interventions	Clinical physiotherapist
Intervention(s) setting	Hospital
Control	Usual care
Outcomes of interest (types and measuring instruments)	Disability - 30-item DASH questionnaire<br>Hand functional ability - Grip Ability Test (GAT)<br>Handgrip strength - hydraulic handgrip<br>Dynamometer<br>Quality of life - 30-item Rheumatoid<br>Arthritis Quality of Life (RAQoL)<br>Confidence - Arthritis<br>Self-Efficacy Scale (ASES)<br>DAS28-ESR<br>Pain – VAS<br>Fatigue - VAS
Methods of analysis	Full factorial mixed-method analysis of variance<br>Post hoc analyses using dependent t-tests with Bonferroni adjustment for multiple comparisons were conducted
Effect size	DASH 12 weeks – 0.50<br>DASH 36 weeks – 0.07
95% Confidence intervals	DASH 12 weeks – 0.50 (0.07-0.93)<br>DASH 36 weeks – 0.07 (-0.35-0.49)
P value	At 12 weeks, there was a significant between-group difference, all favouring the EXTRA programme, in the mean change in<br>• disability (-6.8 [95% confidence interval (95% CI)-12.6,-1.0]; P =0.022),<br>• function (-3.0 [95% CI -5.0, -0.5]; P=0.011),<br>• non-dominant handgrip strength (31.3N [95% CI 9.8,52.8]; P =0.009),<br>• self-efficacy (10.5 [95% CI 1.6, 19.5]; P=0.021<br>• pain 9.3 [95% CI 0.5, 18.2]; P =0.039 for symptoms), and<br>• disease activity (-0.7 [95% CI -1.4, 0.0]; P =0.047)
Follow-up	9 months
Conclusions	The EXTRA program improves upper extremity disability, function, handgrip strength, and self-efficacy in people with RA, with no adverse effects on disease activity.

Niedermann et al., 2011 & Niedermann et al., 2012	
Participants characteristics (number, age, disease criteria, details)	Early RA patients (n=54)<br>≈ 58 years<br>≈ Disease duration 9.3 years<br>ACR Criteria
Intervention(s)	Pictorial Representation of Illness and Self Measure (PRISM) is an interactive hands-on-tool, assessing (a) the individual's perceived burden of illness and (b) relevant individual resources
Intervention(s) characteristics	The PRISM-JP education consisted individualized education and is based on the PRISM tasks of social learning and self-management.<br>The programme consists in 4, 45-min sessions within 3 weeks.<br>In session 1, the standard PRISM task was used to assess perceived burden of illness caused by the RA or pain and identify individual goals.<br>In session 2, The PRISM+ task helped to find the most important individual resource. Patients were asked what activities were most important to them.<br>In sessions 3 and 4, the selected resource was evaluated and reinforced.
Professional that promoted the interventions	Occupational therapists
Intervention(s) setting	Hospital
Control	Conventional JP education
Outcomes of interest (types and measuring instruments)	Joint protection behaviour - Joint Protection Behaviour Assessment D-JPBA-S, Psychological status - Arthritis Self-efficacy Scale, German Version (ASES-D); Hospital Anxiety and Depression Scale, German Version, (HADS-D), Hand status - Grip strength using Jamar hand, Dynamometer; hand pain (VAS scale); DAS28, Quality of life - EUROHIS-QUOL 8
Methods of analysis	Unpaired t-tests for between group comparisons, paired t-tests for within-group comparisons at 3 months and linear regression analysis. Mann–Whitney U-tests and Wilcoxon signed ranks tests were applied for ordinal data to compare between-groups and within-groups, respectively. The relationship between change of primary outcome variable and predictor variables was analysed by linear regression analysis
Effect size	Not stated
95% Confidence inter.	Not stated
P value	At 3 months, the PRISM-JP (n = 26) participants did significantly better compared to the C-JP participants (n = 27) in<br>• JP behaviour (p = 0.02 and p = 0.008 when corrected for baseline values),<br>• Arthritis Self-efficacy (ASES, p = 0.015) and<br>• JP self-efficacy (JP-SES, p = 0.047).<br>Within-group analysis also showed less hand pain (p < 0.001) in PRISM-JP group.<br>At 12 months, the PRISM-JP group (n = 26) demonstrated significantly more JP behaviour at six months (effect size ES = 0.32; p = 0.02) and 12 months (ES = 0.28; p = 0.04) than the C-JP (n = 27).<br>Within-group analysis showed that the JP intervention was successful at six and 12 months in both groups (p < 0.001).<br>At 12 months the PRISM-JP group had better JP self-efficacy (p = 0.02) and grip strength (p = 0.04) compared with baseline
Follow-up	3 months<br>12 months
Conclusions	At 3 months, PRISM-JP more effectively supported learning of JP methods, with meaningful occupations, resource activation and self-efficacy acting as important mediators.<br>At 12 months, PRISM-JP was more effective than C-JP in terms of long-term JP behaviour at six and 12 months

Seneca et al., 2015	
Participants characteristics (number, age, disease criteria, details)	Early RA patients (n=51)<br>≈ 68% female<br>≈ 58 years<br>≈ Disease duration 1.2 years<br>ACR/EULAR Criteria
Intervention(s)	12 weeks of exercises partly supervised
Intervention(s) characteristics	Participants underwent six weeks of supervised training with 30 minutes of physical fitness on an exercise bike. Thirty minutes of muscle strength training (legs, shoulders, trunk extensors and flexors).<br>Exercises were repeated three times. Bike training was used as warm-up before strength exercises. The strength exercises were circle training giving a rest period of approximately five minutes between each set. Intensity load was increased at least every two weeks and adjusted to the patients' symptoms. The supervised sessions were held twice a week in groups of 2-4 patients.
Professional that promoted the interventions	Physiotherapist
Intervention(s) setting	Hospital and community
Control	Self-administered exercises
Outcomes of interest (types and measuring instruments)	Muscle strength - Cybex strengthtraining equipment<br>Physical fitness - submaximal cycle test (Astrand test)<br>Pain – Numerical scale<br>DAS28-CRP<br>Health Assessment Questionnaire (HAQ-DI)<br>The Short Form 36 Health Survey (SF36)<br>Anxiety and fear avoidance in relation to physical activity - Fear Advoidance Belief Questionnaire (FABQ);
Methods of analysis	Spearman's rank correlation analyses
Effect size	Not stated
95% Confidence inter.	Not stated
P value	Following the 12 weeks of exercises, patients in the two groups had improved both their muscle strength and their physical fitness. There was a significant difference in Disease Activity Score in 28 joints calculated with C-reactive protein between the two exercise groups, but no significant differences in physical fitness, pain perception, Health Assessment Questionnaire, Short Form 36 health survey questionnaire, Fear-Avoidance Beliefs Questionnaire, or in muscle strength, except from a significant difference in trunk extensors.<br>The dropout was 40% in the supervised group versus 20% in the self-administered group.
Follow-up	3 months
Conclusions	The partly supervised exercise programme with follow-up after 12 weeks does not seem to be more effective than the self-administered exercise programme.

Shearn et al., 1985	
Participants characteristics	RA patients (n=81)<br>≈ 74% female<br>≈ 56 years<br>≈ Disease duration 10 years<br>Criteria – ARA
Intervention(s)	Program to assess the value of stress management and mutual support groups on the morbidity and psychologic health of patients with rheumatoid arthritis.
Intervention(s) characteristics	Groups met for 10 weekly 90-minute sessions. The aims of stress management were to help the patient identify sources of stress as well as to learn relaxation techniques and strategies for coping.<br>In mutual support groups the aim was to enhance self-responsibility, exchange information, build relationships, and attempt to decrease social isolation.
Professional that promoted the interventions	Psychologist
Intervention(s) setting	Not stated - Kaiser Permanente Medical Center
Control	Standard care
Outcomes of interest (types and measuring instruments)	Pain<br>Duration of morning stiffness<br>ESR<br>Grip strength<br>Walking speed<br>Number of tender joints<br>Disability<br>Life satisfaction<br>Depression – CES- D Scale
Methods of analysis	T test; Analyses of covariance was performed to adjust for the effects of age, sex, disease duration, and pre-test scores on change in the outcome measures.
Effect size	Not stated
95% Confidence inter.	Not stated
P value	Of all these physiologic components, only tender joints showed significant improvement at four months ($p < 0.05$).<br>The psychologic measures of depression and life satisfaction showed no significant change for either intervention group or for the combined intervention groups compared with the control group.<br>No outcome measure showed a significant change from those recorded before any intervention.
Follow-up	4 months
Conclusions	Patients in the Intervention groups showed greater improvement in joint tenderness than in the control patients but did not differ significantly from the patients in the control group in any of the other outcome measures.

Shigaki et al., 2013	
Participants characteristics	RA patients (n=168)<br>≈ 92.5% female<br>≈ 50 years<br>≈ Disease duration 8 years<br>Criteria – not stated
Intervention(s)	Self-management program in rheumatoid arthritis (RA) using an online, cognitive–behavioral, self-management group program (RAHelp), with weekly telephone support
Intervention(s) characteristics	A secure web site (RAHelp.org) to provide a 10-week program with weekly educational modules. A “homework” journal was provided with self-monitoring tools for members to track pain and stress Text boxes were also provided, where pleasant events and weekly challenges could be described. In addition to online features, each member was provided with 1:1 leader support through weekly phone contacts, typically lasting between 15 and 30 minutes. Members had access to several “community” features and activities. Each member created a structured profile and selected an avatar, made available to other members.
Professional that promoted the interventions	Clinicians
Intervention(s) setting	Not stated - Internet
Control	Waiting list control group
Outcomes of interest (types and measuring instruments)	Arthritis Impact Measurement Scales 2 (AIMS2)<br>Arthritis Self-Efficacy Scale (ASES)<br>Center for Epidemiologic Studies Depression Scale (CES-D)<br>Quality of Life Scale (QLS)<br>Rapid Assessment of Disease Activity in Rheumatology (RADAR)<br>Social Provisions Scale (SPS)<br>University of California, Los Angeles Loneliness Scale, version 3 (LS-3).
Methods of analysis	Wilcoxon rank sum test<br>Nonparametric analysis of covariance
Effect size	Effect sizes remained large for self-efficacy and moderate for quality of life.
95% Confidence inter.	Not stated
P value	Group differences with large and moderate effect sizes (ES) were found immediately postintervention for self-efficacy (ASES; ES 0.92, P< 0.00001) and quality of life (QLS; ES 0.66, P < 0.003), respectively.<br>At 9 months postintervention, differences in self-efficacy (ASES; ES 0.92, P <0.00001) and quality of life (QLS; ES 0.71, P < 0.004) remained robust.
Follow-up	9 months
Conclusions	RAHelp appears to have beneficial effects in terms of self-efficacy and quality of life among individuals with RA who are willing to use an online service format.

van Lankveld et al., 2004	
Participants characteristics (number, age, disease criteria, details)	Couples with a patient with RA (n=59)<br>≈ 65% female<br>≈ 50 years<br>≈ 7.2 years disease duration<br>Disease criteria – ACR criteria 1987
Intervention(s)	Cognitive-behavioural oriented self-management treatment with both spouse
Intervention(s) characteristics	The program combines education with CBT. The goal is to restructure disease related cognitions and to teach effective (active) coping styles using rational emotive therapy (RET). Cognitions targeted are the most important stressors of the disease: pain, limitations, and dependence. Patients meet for 8 sessions of 1.5 hour. One session focused on information-giving. In 3 sessions patients are educated about the treatment of RA. The remaining 4 lessons focus on changing the patient's cognitions and behaviour by using RET.
Professional that promoted the interventions	Rheumatologist, psychologist, nurse, nutritionist, physiotherapist, occupational therapist, and social worker
Intervention(s) setting	Sint Maartenskliniek- hospital
Control	Patients without spouse
Outcomes of interest (types and measuring instruments)	DAS28_ESR<br>Physical functioning - Impact of Rheumatic Diseases on General Health and Lifestyle instrument (IRGL)<br>Psychological functioning – IRGL<br>Cognitive evaluation of disease stressors – pain with IRGL; Perceived limitations and dependence (Likert scales)<br>Patient's passive pain coping - Coping with Rheumatoid Stressors (CORS)<br>Marital satisfaction - Marital Satisfaction of the Maudsley Marital Questionnaire (MMQ)<br>Social support – IRGL<br>Spousal criticism - spouse reaction questionnaire<br>Communication improvement – developed a scale
Methods of analysis	General linear model for repeated measures
Effect size	Not stated
95% Confidence inter.	Not stated
P value	In both conditions, similar positive changes in disease activity, cognitions, coping, and physical and psychological functioning were observed. Patients reported a decrease in potential support. There were no differences between conditions. However, at the follow up assessment patients in the experimental condition reported more improvement of disease related communication with their spouse.
Follow-up	6 months
Conclusions	No evidence was found for additional beneficial effects of spouse participation in the cognitive-behavioural oriented self-management group treatment.

Zuidema et al 2019	
Participants characteristics (number, age, disease criteria, details)	RA patients (n=157)<br>≈ 66% female<br>≈ 62 years<br>≈ Disease duration (median control 17 years/median intervention 9 years)<br>Disease criteria – not stated
Intervention(s)	Web-Based Self-Management Enhancing Program
Intervention(s) characteristics	Program comprises 9 modules with 13 performance objectives and a diary to track patients' fatigue and pain over time. Each module comprises 2-5 sessions, with informational and persuasive texts, videos with instructions and role models, exercises, and assignments. The program is unguided, and patients need to choose a module by their own and can work through it at their own pace whenever they want
Professional that promoted the interventions	The program was promoted by nurses although is not clear the professionals that perform intervention
Intervention(s) setting	Specialized hospital in rheumatology, rehabilitation, and orthopaedic surgery
Control	Usual care
Outcomes of interest (types and measuring instruments)	Self-management behaviour -Patient Activation Measurement (PAM-13); Self-Management Ability Scale (SMAS-S))<br>Self-efficacy - Self-Efficacy (RASE), Perceived Efficacy in Patient-Physician Interaction (PEPPI-5)<br>General health status - RAND-36<br>Coping with fatigue - Modified Pain Coping Inventory for Fatigue (MPCI-F)<br>Level of pain and fatigue - NRS
Methods of analysis	Linear mixed model
Effect size	Effect sizes were low.
95% Confidence inter.	Not stated
P value	No positive effects were found regarding the outcome measurements
Follow-up	12 months
Conclusions	It was not possible to conclude on the positive effects of the intervention or to select outcome measures to be regarded as the primary/main or secondary outcomes for a future trial. A process evaluation should be performed to provide more insight into the low compliance with and effectiveness of the intervention
