# Supplementary material for: Effectiveness of self-management interventions in inflammatory arthritis: a systematic review informing the 2021 EULAR recommendations for the implementation of self-management strategies in patients with inflammatory arthritis
Source: RMD Open. 2021 May 28;7(2):e001647. doi: 10.1136/rmdopen-2021-001647 (PMC8166594; doi:10.1136/rmdopen-2021-001647)
Supplement: Supplementary data [file rmdopen-2021-001647supp005.pdf]

Online supplementary material S5: GRADE Summary of Findings

GRADE Summary of Findings

Self-management in inflammatory arthritis

Cognitive Behavior Therapy

Patients or population: Adult patients with rheumatoid arthritis, ankylosing spondylitis, early inflammatory arthritis or psoriatic arthritis

Setting: Self-help organization, hospital, online platform

Comparison: Waiting list, arthritis education, patients without spouse, fatigue information alone, standard programme, usual care

Outcomes	Impact	№ of participants (studies)	Certainty of the evidence (GRADE)
**Functional disability**<br>Several instruments were used to measure this outcome	Effective short and long-term improvements with small positive effects*	633 patients (6 RCTs)	⊕⊕◯◯<br>LOW<br>due to inconsistency (due to significant heterogeneity) and imprecision (due to non-significant effect sizes)
**Disease activity**<br>Several instruments were used to measure this outcome	Effective short and long-term improvements with small positive effects*	466 patients (4 RCTs)	⊕⊕◯◯<br>LOW<br>due to inconsistency (due to significant heterogeneity) and imprecision (due to non-significant effect sizes)
**Impairment/ disability**<br>Several instruments were used to measure this outcome	Effective short and long-term improvements with small positive effects*	165 patients (2 RCTs)	⊕⊕⊕◯<br>MODERATE<br>due to inconsistency (due to significant heterogeneity)
**Anxiety/ depression**<br>Several instruments were used to measure this outcome	Effective short and medium improvements with moderate positive effects**	392 patients (4 RCTs)	⊕⊕◯◯<br>LOW<br>due to inconsistency (due to significant heterogeneity) and imprecision (due to non-significant effect sizes)
**Psychophysiological complains**<br>Several instruments were used to measure this outcome	Effective short and long-term improvements with small to moderate positive effects**	660 patients (6 RCTs)	⊕⊕◯◯<br>LOW<br>due to inconsistency (due to significant heterogeneity) and imprecision (due to non-significant effect sizes)
**Sleep problems**<br>Several instruments were used to measure this outcome	Effective short-term improvement with moderate positive effects**	165 patients (2 RCTs)	⊕⊕◯◯<br>LOW<br>due to inconsistency (due to significant heterogeneity) and imprecision (due to non-significant effect sizes)
**Pain**<br>Several instruments were used to measure this outcome	Effective short and long-term improvements with small positive effects*	560 patients (5 RCTs)	⊕⊕◯◯<br>LOW<br>due to inconsistency (due to significant heterogeneity) and imprecision (due to non-significant effect sizes)

**Self-efficacy/ self-helplessness**<br>Several instruments were used to measure this outcome	Effective short and long-term improvements with small to moderate positive effects**	461 patients (3 RCTs)	⨁⨁⨁◯<br>MODERATE<br>due to inconsistency (due to significant heterogeneity)
**Quality of life/ health status/ social support**<br>Several instruments were used to measure this outcome	Effective long-term improvement with positive effects	353 patients (3 RCTs)	⨁⨁◯◯<br>LOW<br>due to inconsistency (due to significant heterogeneity) and imprecision (due to non-significant effect sizes)
**Health care use**<br>Several instruments were used to measure this outcome	No effect or difference compared to a control treatment	167 patients (1 RCT)	⨁⨁◯◯<br>LOW<br>due to inconsistency (due to significant heterogeneity) and imprecision (due to non-significant effect sizes)
**Fatigue**<br>Several instruments were used to measure this outcome	Effective short and long-term improvements with small to moderate positive effects**	352 patients (3 RCTs)	⨁⨁⨁◯<br>MODERATE<br>due to inconsistency (due to significant heterogeneity)

Response training

Patients or population: Adult patients with rheumatoid arthritis or early rheumatoid arthritis

Setting: Hospital

Comparison: Usual care or arthritis education

Outcomes	Impact	№ of participants (studies)	Certainty of the evidence (GRADE)
**Functional disability**<br>Several instruments were used to measure this outcome	Effective short and long-term improvements with small to moderate positive effects**	276 patients (2 RCTs)	⨁⨁⨁◯<br>MODERATE<br>due to inconsistency (due to significant heterogeneity)
**Disease activity**<br>Several instruments were used to measure this outcome	Effective short and long-term improvements with small to moderate positive effects**	276 patients (2 RCTs)	⨁⨁⨁◯<br>MODERATE<br>due to inconsistency (due to significant heterogeneity)
**Impairment/ disability**<br>Several instruments were used to measure this outcome	Effective long-term improvement with small to moderate positive effects**	108 patients (1 RCT)	⨁⨁⨁◯<br>MODERATE<br>due to inconsistency (due to significant heterogeneity)
**Psychophysiological complains**<br>Several instruments were used to measure this outcome	No effect or difference compared to a control treatment	168 patients (1 RCT)	⨁⨁◯◯<br>LOW<br>due to inconsistency (due to significant heterogeneity) and imprecision (due to non-significant effect sizes)
**Pain**<br>Several instruments were used to measure this outcome	Effective short and long-term improvements with small to moderate positive effects**	276 patients (2 RCTs)	⨁⨁⨁◯<br>MODERATE<br>due to inconsistency (due to significant heterogeneity)
**Self-Efficacy/ self-helplessness**<br>Several instruments were used to measure this outcome	Effective short and long-term improvements with small to moderate positive effects**	108 patients (1 RCT)	⨁⨁⨁◯<br>MODERATE<br>due to inconsistency (due to significant heterogeneity)

**Quality of life/Health status/Social support**<br>Several instruments were used to measure this outcome	Effective short and long-term improvements with small to moderate positive effects**	108 patients (1 RCT)	⊕⊕⊕◯<br>MODERATE<br>due to inconsistency (due to significant heterogeneity)
**Fatigue**<br>Several instruments were used to measure this outcome	Effective short and long-term improvements with small to moderate positive effects**	108 patients (1 RCT)	⊕⊕⊕◯<br>MODERATE<br>due to inconsistency (due to significant heterogeneity)

Specific interactive disease education

Patients or population: Adult patients with rheumatoid arthritis or early rheumatoid arthritis

Setting: Hospital, online platform and community

Comparison: Arthritis education, waiting list, self-administered exercise, control writing+control training, usual care

Outcomes	Impact	№ of participants (studies)	Certainty of the evidence (GRADE)
**Knowledge**<br>Several instruments were used to measure this outcome	Effective long-term improvement with positive effects	208 patients (1 RCT)	⊕⊕⊕◯<br>MODERATE<br>due to inconsistency (due to significant heterogeneity)
**Functional disability**<br>Several instruments were used to measure this outcome	Effective short and long-term improvements with small to moderate positive effects**	1065 patients (8 RCTs)	⊕⊕◯◯<br>LOW<br>due to inconsistency (due to significant heterogeneity) and imprecision (due to non-significant effect sizes)
**Disease activity**<br>Several instruments were used to measure this outcome	Effective short and long-term improvements with small to moderate positive effects**	1065 patients (8 RCTs)	⊕⊕◯◯<br>LOW<br>due to inconsistency (due to significant heterogeneity) and imprecision (due to non-significant effect sizes)
**Impairment/ disability**<br>Several instruments were used to measure this outcome	Effective short and long-term improvements with small to moderate positive effects**	453 patients (3 RCTs)	⊕⊕◯◯<br>LOW<br>due to inconsistency (due to significant heterogeneity) and imprecision (due to non-significant effect sizes)
**Anxiety/ depression**<br>Several instruments were used to measure this outcome	No effect or difference compared to a control treatment	586 patients (5 RCTs)	⊕⊕⊕◯<br>MODERATE<br>due to inconsistency (due to significant heterogeneity)
**Psychophysiological complains**<br>Several instruments were used to measure this outcome	No effect or difference compared to a control treatment	600 patients (3 RCTs)	⊕⊕⊕◯<br>MODERATE<br>due to inconsistency (due to significant heterogeneity)
**Pain**<br>Several instruments were used to measure this outcome	Effective short and long-term improvements with small to moderate positive effects**	997 patients (7 RCTs)	⊕⊕◯◯<br>LOW<br>due to inconsistency (due to significant heterogeneity) and imprecision (due to non-significant effect sizes)
**Self-efficacy/ self-helplessness**<br>Several instruments were used to measure this outcome	Effective short and long-term improvements with small to moderate positive effects**	866 patients (6 RCTs)	⊕⊕◯◯<br>LOW<br>due to inconsistency (due to significant heterogeneity) and imprecision (due to non-significant effect sizes)

**Quality of life/ health status/ social support**<br>Several instruments were used to measure this outcome	Effective short and long-term improvements with small to moderate positive effects**	788 patients (5 RCTs)	⊕⊕◯◯<br>LOW<br>due to inconsistency (due to significant heterogeneity) and imprecision (due to non-significant effect sizes)
**Fatigue**<br>Several instruments were used to measure this outcome	Effective short and long-term improvements with small positive effects*	551 patients (4 RCTs)	⊕⊕◯◯<br>LOW<br>due to inconsistency (due to significant heterogeneity) and imprecision (due to non-significant effect sizes)

Goal setting

Patients or population: Adult patients with rheumatoid arthritis or early rheumatoid arthritis

Setting: Hospital

Comparison: Arthritis education, education, fatigue information alone, usual care

Outcomes	Impact	№ of participants (studies)	Certainty of the evidence (GRADE)
**Functional disability**<br>Several instruments were used to measure this outcome	Effective short and long-term improvements with small to moderate positive effects**	632 patients (6 RCTs)	⊕⊕◯◯<br>LOW<br>due to inconsistency (due to significant heterogeneity) and imprecision (due to non-significant effect sizes)
**Disease activity**<br>Several instruments were used to measure this outcome	Effective short and long-term improvements with small to moderate positive effects**	452 patients (4 RCTs)	⊕⊕◯◯<br>LOW<br>due to inconsistency (due to significant heterogeneity) and imprecision (due to non-significant effect sizes)
**Impairment / disability**<br>Several instruments were used to measure this outcome	Effective short-term improvement with moderate positive effects**	126 patients (1 RCT)	⊕⊕⊕◯<br>MODERATE<br>due to inconsistency (due to significant heterogeneity)
**Anxiety/ depression**<br>Several instruments were used to measure this outcome	Effective short and long-term improvements with small to moderate positive effects**	317 patients (4 RCTs)	⊕⊕◯◯<br>LOW<br>due to inconsistency (due to significant heterogeneity) and imprecision (due to non-significant effect sizes)
**Psychophysiological complains**<br>Several instruments were used to measure this outcome	Effective short-term improvement with small positive effects*	281 patients (3 RCTs)	⊕⊕◯◯<br>LOW<br>due to inconsistency (due to significant heterogeneity) and imprecision (due to non-significant effect sizes)
**Sleep problems**<br>Several instruments were used to measure this outcome	Effective short-term improvement with moderate positive effects**	126 patients (1 RCT)	⊕⊕⊕◯<br>MODERATE<br>due to inconsistency (due to significant heterogeneity)
**Pain**<br>Several instruments were used to measure this outcome	Effective short and long-term improvements with small positive effects*	227 patients (2 RCTs)	⊕⊕◯◯<br>LOW<br>due to inconsistency (due to significant heterogeneity) and imprecision (due to non-significant effect sizes)

**Self-efficacy/ self-helplessness**<br>Several instruments were used to measure this outcome	Effective short-term improvement with small positive effects*	405 patients (4 RCTs)	⊕⊕◯◯<br>LOW<br>due to inconsistency (due to significant heterogeneity) and imprecision (due to non-significant effect sizes)
**Quality of life/ health status/ social support**<br>Several instruments were used to measure this outcome	Effective short and long-term improvements with small positive effects*	327 patients (3 RCTs)	⊕⊕◯◯<br>LOW<br>due to inconsistency (due to significant heterogeneity) and imprecision (due to non-significant effect sizes)
**Fatigue**<br>Several instruments were used to measure this outcome	Effective short and long-term improvements with moderate positive effects**	263 patients (3 RCTs)	⊕⊕◯◯<br>LOW<br>due to inconsistency (due to significant heterogeneity) and imprecision (due to non-significant effect sizes)

Problem solving

Patients or population: Adult patients with rheumatoid arthritis, ankylosing spondylitis, early inflammatory arthritis or psoriatic arthritis

Setting: Hospital and online platform

Comparison: Arthritis education, waiting list, education, fatigue information alone, control writing+control training, usual care

Outcomes	Impact	№ of participants (studies)	Certainty of the evidence (GRADE)
**Functional disability**<br>Several instruments were used to measure this outcome	Effective short and long-term improvements with small to moderate positive effects**	1063 patients (8 RCTs)	⊕⊕◯◯<br>LOW<br>due to inconsistency (due to significant heterogeneity) and imprecision (due to non-significant effect sizes)
**Disease activity**<br>Several instruments were used to measure this outcome	Effective short and long-term improvements with small to moderate positive effects**	716 patients (5 RCTs)	⊕⊕◯◯<br>LOW<br>due to inconsistency (due to significant heterogeneity) and imprecision (due to non-significant effect sizes)
**Impairment/ disability**<br>Several instruments were used to measure this outcome	Effective short improvement with moderate positive effects**	390 patients (2 RCTs)	⊕⊕◯◯<br>LOW<br>due to inconsistency (due to significant heterogeneity) and imprecision (due to non-significant effect sizes)
**Anxiety/ depression**<br>Several instruments were used to measure this outcome	Effective short and long-term improvements with small to moderate positive effects**	485 patients (5 RCTs)	⊕⊕◯◯<br>LOW<br>due to inconsistency (due to significant heterogeneity) and imprecision (due to non-significant effect sizes)
**Psychophysiological complains**<br>Several instruments were used to measure this outcome	Effective short and long-term improvements with small to moderate positive effects**	880 patients (6 RCTs)	⊕⊕◯◯<br>LOW<br>due to inconsistency (due to significant heterogeneity) and imprecision (due to non-significant effect sizes)
**Sleep problems**<br>Several instruments were used to measure this outcome	Effective short improvement with moderate positive effects**	126 patients (1 RCT)	⊕⊕⊕◯<br>MODERATE<br>due to inconsistency (due to significant heterogeneity)

**Pain**<br>Several instruments were used to measure this outcome	Effective short and long-term improvements with small positive effects*	826 patients (5 RCTs)	⊕⊕◯◯<br>LOW<br>due to inconsistency (due to significant heterogeneity) and imprecision (due to non-significant effect sizes)
**Self-efficacy/ self-helplessness**<br>Several instruments were used to measure this outcome	Effective short and long-term improvements with small to moderate positive effects**	740 patients (6 RCTs)	⊕⊕◯◯<br>LOW<br>due to inconsistency (due to significant heterogeneity) and imprecision (due to non-significant effect sizes)
**Quality of life/ health status/ social support**<br>Several instruments were used to measure this outcome	Effective short and long-term improvements with small to moderate positive effects**	495 patients (4 RCTs)	⊕⊕◯◯<br>LOW<br>due to inconsistency (due to significant heterogeneity) and imprecision (due to non-significant effect sizes)
**Health care use**<br>Several instruments were used to measure this outcome	No effect or difference compared to a control treatment	167 patients (1 RCT)	⊕⊕⊕◯<br>MODERATE<br>due to inconsistency (due to significant heterogeneity)
**Fatigue**<br>Several instruments were used to measure this outcome	Effective short and long-term improvements with small to moderate positive effects**	430 patients (4 RCTs)	⊕⊕◯◯<br>LOW<br>due to inconsistency (due to significant heterogeneity) and imprecision (due to non-significant effect sizes)

Multicomponent or single exercise / physical activity interventions

Patients or population: Adult patients with rheumatoid arthritis and ankylosing spondylitis

Setting: Home, hospitals, spas, pools, fitness centres

Interventions: Multi-component exercises involving resistance, strengthening, aerobic exercises and flexibility or single modalities of exercise; yoga; Tai Chi; specific aquatic exercises (e.g. swimming)

Comparison: No exercise, usual care, non-aerobic exercises, range of motion exercises or education No exercise, usual care, non-aerobic exercises, range of motion exercises or education, alternative exercise method, home exercise regimes

Outcomes	Impact	№ of participants (studies)	Certainty of the evidence (GRADE)
**Pain**<br>Several instruments were used to measure this outcome	Effective improvement with small positive effects*	58 RCTs (5 reviews)	⊕⊕⊕◯<br>MODERATE<br>due to inconsistency (due to significant heterogeneity)
**Functional disability**<br>Several instruments were used to measure this outcome	Effective improvement with small positive effects*	26 RCTs (3 reviews)	⊕⊕◯◯<br>LOW<br>due to inconsistency (due to significant heterogeneity) and imprecision (due to non-significant effect sizes)
**Fatigue**<br>Several instruments were used to measure this outcome	Effective improvement with small positive effects*	24 RCTs (1 review)	⊕⊕⊕⊕<br>HIGH
**Patient Global Assessment**	Effective improvement with small positive effects*	11 RCTs (1 review)	⊕⊕⊕◯<br>MODERATE<br>due to inconsistency (due to significant heterogeneity)

**BASDAI**	Effective improvement with small positive effects*	16 RCTs (2 reviews)	⨁⨁◯◯<br>LOW<br>due to inconsistency (due to significant heterogeneity) and publication bias (due to not having been being assessed)
**BASFI**	Effective improvement with small positive effects*	16 RCTs (2 reviews)	⨁⨁◯◯<br>LOW<br>due to inconsistency (due to significant heterogeneity) and publication bias (due to not having been being assessed)
**DAS-28**	No effect or difference compared to a control treatment	7 RCTs (1 review)	⨁◯◯◯<br>VERY LOW<br>due to inconsistency (due to significant heterogeneity), imprecision (due to non-significant effect sizes) and publication bias (due to not having been being assessed

Psychosocial interventions

Patients or population: Adult patients with inflammatory arthritis [rheumatoid arthritis (mainly), psoriatic arthritis and ankylosing spondylitis]

Setting: Home, fitness centres (majority not stated)

Interventions: Expressive writing, cognitive skills training, cognitive behavioral interventions which involve the combination of relaxation, biofeedback, imagery, stress management, or the teaching of cognitive coping skills; mindfulness-based interventions, lifestyle management, patient education incorporating energy conservation, self-management interventions, group education, counselling, psychotherapy, relaxation, cognitive pain management strategies, contracting, goal setting, provision of feedback, cognitive restructuring, joint protection, problem solving, guided imagery, self-instruction

Comparison: Usual medical care, social support, support group, symptom monitoring wait list control, occupational therapy, education, or no intervention

Outcomes	Impact	№ of participants (studies)	Certainty of the evidence (GRADE)
**Pain**<br>Several instruments were used to measure this outcome	Effective short and long-term improvements with small to moderate positive effects**	88 RCTs (4 reviews)	⨁⨁⨁◯<br>MODERATE<br>due to inconsistency (due to significant heterogeneity)
**Functional disability**<br>Several instruments were used to measure this outcome	Effective short-term improvement with small positive effects*	83 RCTs (3 reviews)	⨁⨁⨁◯<br>MODERATE<br>due to inconsistency (due to significant heterogeneity)
**Fatigue**<br>Several instruments were used to measure this outcome	Effective improvement with small positive effects*	24 RCTs (1 review)	⨁⨁⨁◯<br>MODERATE<br>due to inconsistency (due to significant heterogeneity)
**Psychological status**<br>Several instruments were used to measure this outcome	Effective short and long-term improvements with small positive effects*	25 RCTs (1 review)	⨁⨁⨁◯<br>MODERATE<br>due to inconsistency (due to significant heterogeneity)
**Physical activity**<br>Several instruments were used to measure this outcome	Effective short and long-term improvements with small to moderate positive effects**	27 RCTs (1 review)	⨁⨁⨁◯<br>MODERATE<br>due to inconsistency (due to significant heterogeneity)

**Depression** Several instruments were used to measure this outcome	Effective short and long-term improvements with small positive effects*	27 RCTs (1 review)	⊕⊕⊕◯ MODERATE due to inconsistency (due to significant heterogeneity)
**Anxiety** Several instruments were used to measure this outcome	Effective long-term improvements with small positive effects*	27 RCTs (1 review)	⊕⊕⊕◯ MODERATE due to inconsistency (due to significant heterogeneity)
**Tender joints**	Effective long-term improvement with small positive effects*	25 RCTs (1 review)	⊕⊕⊕◯ MODERATE due to inconsistency (due to significant heterogeneity)
**Coping** Several instruments were used to measure this outcome	Effective short and long-term improvements with moderate positive effects**	25 RCTs (1 review)	⊕⊕⊕◯ MODERATE due to inconsistency (due to significant heterogeneity)
**Self-efficacy** Several instruments were used to measure this outcome	Effective short-term improvement with small positive effects*	56 RCTs (2 reviews)	⊕⊕⊕◯ MODERATE due to inconsistency (due to significant heterogeneity)
**DAS-28**	No effect or difference compared to a control treatment	5 RCTs (1 review)	⊕◯◯◯ VERY LOW due to inconsistency (due to significant heterogeneity), imprecision (due to non-significant effect sizes) and publication bias (due to not having been being assessed)

Self-management interventions

Patients or population: Adult patients with inflammatory arthritis (rheumatoid arthritis and psoriatic arthritis)

Setting: Home, although the majority not stated it

Interventions: Self-management interventions (including swimming sessions, relaxation, exercises, low impact land-based exercises, sessions on activities of daily living and education-discussion sessions, walking and Tai Chi)

Comparison: Waiting-list control, education booklet, usual care, standard programs, no treatment

Outcomes	Impact	№ of participants (studies)	Certainty of the evidence (GRADE)
**Pain** Several instruments were used to measure this outcome	Effective short and long-term improvements with small positive effects*	19 RCTs (1 review)	⊕⊕◯◯ LOW due to inconsistency (due to significant heterogeneity) and publication bias (due to not having been being assessed)
**Functional disability** Several instruments were used to measure this outcome	No effect or difference compared to a control treatment	19 RCTs (1 review)	⊕◯◯◯ VERY LOW due to inconsistency (due to significant heterogeneity), imprecision (due to non-significant effect sizes) and publication bias (due to not having been being assessed)

Educational interventions

Patients or population: Adult patients with rheumatoid arthritis

Setting: Hospital

Interventions: Educational Interventions, information only, leaflets, counselling, behavioural treatment, joint protection and energy conservation, disease education, range of motion and strengthening, physical agent modalities, cognitive rehabilitation, environmental adaptation, provision of adaptive equipment, maintaining activities

Comparison: Symptom monitoring, no-intervention, waiting list controls, standard rheumatologic care, lectures, other leaflets, or education

Outcomes	Impact	№ of participants (studies)	Certainty of the evidence (GRADE)
**Pain**<br>Several instruments were used to measure this outcome	Effective improvement with positive effects	22 RCTs (1 review)<br><br>208 patients (2 RCTs)	⊕⊕⊕◯<br>MODERATE<br>due to inconsistency (due to significant heterogeneity)
**Fatigue**<br>Several instruments were used to measure this outcome	Effective improvement with positive effects	22 RCTs (1 review)<br><br>78 patients (1 RCT)	⊕◯◯◯<br>VERY LOW<br>due to inconsistency (due to significant heterogeneity), imprecision (due to non-significant effect sizes) and publication bias (due to not having been being assessed)
**Functional disability**<br>Several instruments were used to measure this outcome	Effective short-term improvement with small positive effects*	50 RCTs (1 review<br><br>137 patients (2 RCTs)	⊕⊕⊕◯<br>MODERATE<br>due to inconsistency (due to significant heterogeneity)
**Joint counts**	Effective short-term improvement with small positive effects*	50 RCTs (1 review)	⊕⊕⊕◯<br>MODERATE<br>due to inconsistency (due to significant heterogeneity)
**Patient Global Assessment**	Effective short-term improvement with small positive effects*	50 RCTs (1 review)	⊕⊕⊕◯<br>MODERATE<br>due to inconsistency (due to significant heterogeneity))
**Psychological status**<br>Several instruments were used to measure this outcome	Effective short-term improvement with small positive effects*	50 RCTs (1 review)	⊕⊕⊕◯<br>MODERATE<br>due to inconsistency (due to significant heterogeneity)
**Depression**<br>Several instruments were used to measure this outcome	Effective short-term improvement with small positive effects*	50 RCTs (1 review)<br><br>245 patients (3 RCTs)	⊕⊕⊕◯<br>MODERATE<br>due to inconsistency (due to significant heterogeneity)
**Adherence**<br>Measured by pharmacological marker	Effective improvement with positive effects	100 patients (1 RCTs)	⊕⊕⊕◯<br>MODERATE<br>due to inconsistency (due to significant heterogeneity)
**Self-efficacy**<br>Several instruments were used to measure this outcome	Effective short and long-term improvement with small positive effects*	132 patients (2 RCTs)	⊕⊕⊕◯<br>MODERATE<br>due to inconsistency (due to significant heterogeneity)

*The effect is interpreted as **small positive** because it is less than 0.40
The effect is interpreted as **moderate positive because it is greater than 0.40 and less than 0.80

GRADE Working Group grades of evidence
High certainty: We are very confident that the true effect lies close to that of the estimate of the effect
Moderate certainty: We are moderately confident in the effect estimate: The true effect is likely to be close to the estimate of the effect, but there is a possibility that it is substantially different
Low certainty: Our confidence in the effect estimate is limited: The true effect may be substantially different from the estimate of the effect
Very low certainty: We have very little confidence in the effect estimate: The true effect is likely to be substantially different from the estimate of effect
